# Transdiagnostic efficacy of lurasidone on depressive symptoms: systematic review and meta-analysis of randomised controlled trials

**DOI:** 10.1192/bjp.2025.10343

**Published:** 2026-04

**Authors:** Ana Ghenciulescu, Anna Pearse, Amedeo Minichino, Gaia Sampogna, Riccardo De Giorgi, Andrea Fiorillo

**Affiliations:** Medical Sciences Division, University of Oxford, John Radcliffe Hospital, UK; Oxford Health NHS Foundation Trust, Warneford Hospital, Oxford, UK; University Hospitals of North Midlands NHS Trust, Stoke-on-Trent, UK; Department of Psychiatry, University of Oxford, Warneford Hospital, UK; Department of Psychiatry, University of Campania ‘L. Vanvitelli’, Naples, Italy

**Keywords:** Lurasidone, depression, bipolar disorder, schizophrenia, meta-analysis

## Abstract

**Background:**

Lurasidone is a second-generation antipsychotic with antidepressant properties, but its effect on depressive symptoms across diagnostic domains is not known.

**Aims:**

This systematic review aims to synthesise the evidence for the transdiagnostic efficacy of lurasidone in reducing depressive symptoms.

**Method:**

Electronic databases were searched up to October 2024 to identify randomised controlled trials comparing the effects of lurasidone and placebo on depressive symptoms, as measured by any standardised scale, in populations with different psychiatric diagnoses. Acceptability, tolerability and safety were also measured. The Cochrane risk of bias tool was used to assess study quality, and the GRADE tool to evaluate certainty of evidence. A random-effects meta-analysis was performed to estimate standardised mean differences (SMDs, for continuous outcomes) or relative risks (for dichotomous outcomes) with 95% CI.

**Results:**

Fourteen trials met inclusion criteria. Pooled analysis of 5239 participants found lurasidone to be more efficacious than placebo in improving depression scores (SMD −0.26, 95% CI −0.37, −0.15) across multiple diagnoses (including schizophrenia, bipolar disorder and major depressive disorder). Secondary analyses showed better acceptability (relative risk 0.55, 95% CI 0.43, 0.71) and safety (relative risk 0.73, 95% CI 0.58, 0.91) and comparable tolerability (relative risk 0.74, 95% CI 0.54, 1.02) between lurasidone and placebo. The main limitations were the high risk of bias of several included studies and the high heterogeneity observed in our findings.

**Conclusion:**

Lurasidone is a potentially efficacious and safe strategy for reducing depressive symptomatology across a range of psychiatric diagnoses. Further long-term, robust trials employing precision psychiatry methods are needed to support its broader use to target depressive symptoms transdiagnostically.

Depressive symptoms are highly prevalent across subjects with different psychiatric diagnoses, leading to significant morbidity. For instance, one study has estimated that 60.4% of patients with schizophrenia spectrum disorders experience substantial symptoms of depression,^1^ which are correlated with an increased risk of suicide;^2^ proposed aetiological pathways include a phenomenon intrinsic to psychosis or a psychological reaction to the diagnosis and its social consequences.^3^ In individuals with bipolar disorder, the majority of time is spent in the depressive phase of the illness, with depressive symptoms contributing significantly to impaired functioning.^4^ It has been argued that the high degree of comorbidity among mental health conditions may, in part, represent an artefact of the categorical classification system currently utilised in psychiatric practice,^5^ rather than the coexistence of truly distinct diagnostic domains.^6^ The transdiagnostic approach is a novel framework that is gaining popularity in the psychiatric field^7^ – it seeks to re-examine our understanding of mental health beyond conventional diagnostic boundaries,^8^ and may be more appropriate for examining sets of symptoms that occur across diagnostic categories^6^ such as depressive symptoms. Given the theoretical issues raised by the categorical ‘comorbidity’ paradigm,^9^ and the poorer outcomes associated with higher psychiatric symptom burden, the transdiagnostic approach offers a pragmatic alternative to address the complex symptomatology of patients under mental health care.

Lurasidone, a second-generation antipsychotic approved in the UK for the treatment of schizophrenia,^10^ holds potential as a therapeutic option for depressive symptoms across a range of mental health disorders, warranting further exploration. Atypical antipsychotics are already commonly used in bipolar disorder,^11^ and are an important add-on in treatment-resistant depression.^12^ In the USA, lurasidone is also licensed for bipolar depression^13^ following the 2014 PREVAIL trial, which showed that lurasidone monotherapy significantly reduced depression scores compared with placebo in patients with bipolar I disorder experiencing a major depressive episode (standardised mean difference (SMD) 0.51, *P* < 0.001).^14^ While lurasidone is not licensed for the treatment of depressive symptoms in the UK, in certain cases it may be recommended for off-label use in bipolar depression by guidelines based on expert opinion.^15,16^ Similarly to other atypical antipsychotics, lurasidone acts as an antagonist for D2 and 5-HT2A receptors but, relevantly, also has activity on 5-HT1A and 5-HT7 receptor subtypes, which may mediate its putative antidepressant effect.^17^

A recent network meta-analysis (NMA)^18^ of antidepressants and antipsychotics in patients with an acute major depressive episode of bipolar depression found that lurasidone significantly improved depressive symptoms compared with placebo (SMD 0.29, 95% CI 0.14, 0.45). Similarly, numerous placebo- and antipsychotic-controlled trials published to date support the use of lurasidone in patients with schizophrenia. Of note, an NMA^19^ looking at the efficacy of multiple antipsychotics in treating acute exacerbations of schizophrenia showed that lurasidone improved depressive symptoms (SMD −0.20, 95% CI −0.32, −0.09); however, studies in patients with predominantly negative or depressive symptoms at baseline were excluded. While the literature on lurasidone in major depressive disorder (MDD) is sparse, a meta-analysis of monotherapy with antipsychotics^20^ found significant benefits in remission rates (relative risk 1.57, 95% CI 1.26, 1.95) and depression scores (SMD −0.45, 95% CI −0.64, −0.25) compared with placebo in MDD across the included studies. This reduction was driven by lurasidone and quetiapine, while the pooled effects of haloperidol, olanzapine and ziprasidone were not statistically significant.^20^

These previous reviews are in line with the traditional practice of evaluating the effects of one or multiple pharmacological agents on depressive symptoms within a population with a predefined diagnosis.^21^ However, to our knowledge, there has not been any study examining the effects of lurasidone on a given outcome measure across multiple psychiatric illnesses. Considering the high prevalence of depressive symptoms across diagnoses, as well as the multimodal pharmacological profile of lurasidone that underlies its potential dual antipsychotic and antidepressant effect, it is key to establish whether it can reduce depressive symptomatology regardless of diagnostic domain. Therefore, we leverage a transdiagnostic approach to this systematic review and pairwise meta-analysis,^7^ aiming to assess the antidepressant effects of lurasidone by considering studies performed in populations with a variety of psychiatric diagnoses. To our knowledge, this is the first meta-analysis in the field exploring the therapeutic effects of an antipsychotic agent for depressive symptoms across diagnostic boundaries.

## Method

This meta-analysis adhered to PRISMA guidelines (Supplementary materials 1 available at https://doi.org/10.1192/bjp.2025.10343), and the protocol for this review was registered on PROSPERO (reference ID: CRD42023469284; https://www.crd.york.ac.uk/PROSPERO/view/CRD42023469284). No ethics approval or informed consent were required. Changes from the protocol are summarised in Supplementary materials 11.

### Search strategy

We conducted an extensive search of the databases MEDLINE, Embase and PsycInfo via Ovid, as well as clinical trials registers, including Cochrane CENTRAL, ClinicalTrial.gov, IRCTN and the World Health Organization (WHO) portal, from inception to October 2024, with no restrictions on language or publication period (Supplementary materials 2). Search results were supplemented by a manual screening of references of the included studies and other relevant articles.

### Inclusion criteria

We included all randomised, double-masked, placebo-controlled trials of lurasidone with a primary or secondary efficacy outcome measuring depressive symptoms (Supplementary materials 3). Cluster or quasi-randomised studies and those with unmasked designs were excluded. For studies with a crossover design, we planned to use only data from the first phase before crossover, to avoid carry-over effects. We included participants of both genders, aged 12 years or older and with any diagnosed mental disorder.

### Outcomes

Our primary efficacy outcome was the mean change from baseline to study end-point in the severity of depressive symptoms, measured using the Montgomery–Asberg Depression Rating Scale (MADRS) or any other validated scale for assessing depressive symptom severity. Priority was given to MADRS if multiple depression measures were reported. Our secondary outcomes included:

- acceptability – the proportion of treatment discontinuations due to any cause
- tolerability – the proportion of treatment discontinuations due to any adverse event
- safety – the proportion of participants experiencing at least one adverse event and the type of adverse events experienced by participants.

### Study selection, data extraction, quality and certainty assessment

Two reviewers (A.P., A.G.) independently screened all titles and abstracts identified in the searches, followed by relevant full-text articles for eligibility.

Disagreements were resolved by discussion with a third reviewer (R.D.G.) to reach a consensus. Reasons for exclusion were recorded. Duplicate reports were identified and excluded, while multiple reports related to the same trial were collated. The software Covidence for macOS (Veritas Health Innovation, Melbourne, Australia; www.covidence.org) was used in the data selection process. Two reviewers (A.P., A.G.) independently extracted relevant data from selected studies into standardised spreadsheets, starting 28 February 2024, and then revised these following the updated search in October 2024. These included data on patient characteristics, intervention and comparator details and primary and secondary outcome results, with the time points reported. Extracted data are reported in full in Supplementary materials 13A, B.

The Cochrane risk of bias tool 2 (RoB2)^22^ was used to appraise the risk of bias for the primary outcome. Two reviewers (A.P., A.G.) independently assessed RoB2 in the five domains, followed by assessment of overall risk of bias for each study. Where the information available for studies was insufficient, we attempted to contact authors to gather more information. Disagreements were resolved through discussion with another reviewer (R.D.G.). Certainty of evidence for all outcomes was evaluated using the Grading of Recommendations, Assessment, Development, and Evaluations (GRADE; GRADEpro Guideline Development Tool, McMaster University and Evidence Prime; www.gradepro.org) system (Supplementary materials 10).

### Statistical analysis

Extracted data were analysed using Stata version 17 for macOS. Efficacy data from depression rating scales were analysed as a continuous variable using SMD, because different rating scales were used, with 95% CI, employing a random-effect model, which is more conservative than a fixed-effect model, considering the heterogeneity of the study populations included. In evaluating the clinical significance of SMD values, effect size was considered ’small’ if SMD < 0.40, ‘moderate’ if SMD was 0.40–0.70 and ‘large’ if SMD > 0.70, in keeping with Cochrane guidance.^23^ All other quantitative data (e.g. number of participants discontinuing treatment) were analysed as dichotomous variables using relative risk with 95% CI, using random-effect models. For dropouts and adverse events, zero events of both arms in any trial were replaced by 0.5.^23^ Heterogeneity between studies was investigated through the *I* ^2^, *t* ^2^ and *P*-value statistics, and by visual inspection of the forest plots; 95% prediction intervals were calculated following the Cochrane Handbook formula for random-effect meta-analyses.^23^ Funnel plots and Egger’s test were used to detect publication bias. Sensitivity and subgroup analyses were conducted post hoc to verify the robustness of the primary efficacy findings (Supplementary materials 5, 6).

## Results

### Literature search

**Fig. 1:**
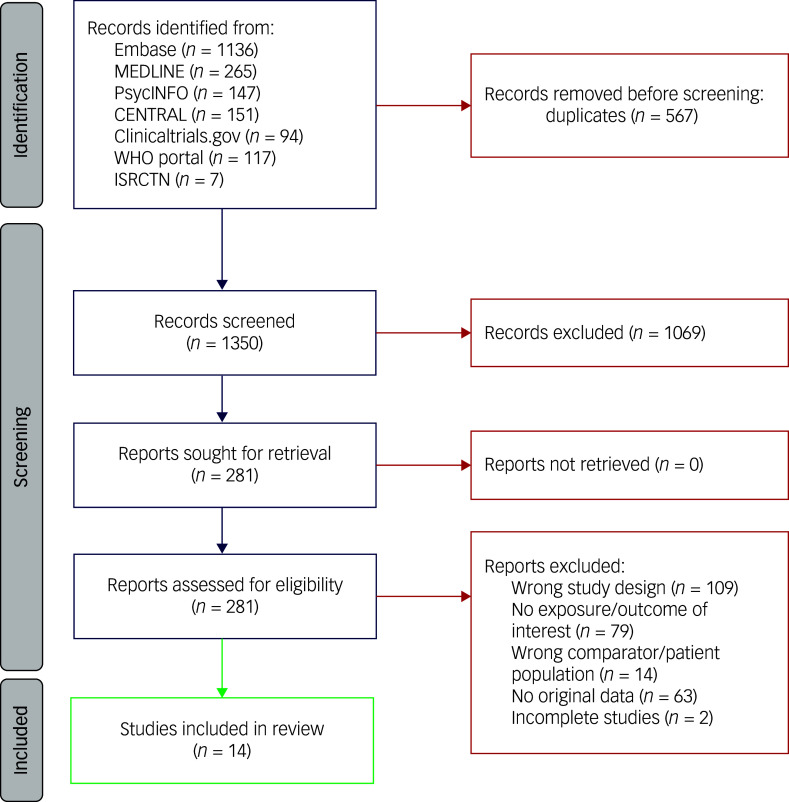
PRISMA 2020 flow diagram. WHO, World Health Organization.

The database search yielded 1917 articles: 1136 in Embase, 265 in PubMed/MEDLINE, 147 in PsycInfo, 151 in Cochrane CENTRAL, 94 in ClinicalTrials.gov, 117 in World Health Organization (WHO) and 7 in ISRCTN. Follwing removal of duplicates, 1350 titles and abstracts were screened according to the above criteria, of which 1069 were excluded due to lack of relevance. Finally, 281 articles were assessed in full for eligibility, of which 14 randomised controlled trials (RCTs) were included in the meta-analysis. A flowchart of the literature search is depicted according to the PRISMA 2020 guidelines in Fig. 1.

### Trial characteristics

Table 1 summarises the characteristics of the 14 included studies^14,24–36^ (Supplementary materials 4). The study samples included patients with schizophrenia experiencing an acute psychotic exacerbation,^26,28,30–34^ patients with bipolar I disorder,^14,24,25,27,29,35^ and patients with MDD with mixed features.^36^ Of the studies investigating bipolar disorder, five of six included only patients with a current major depressive episode.^14,25,27,29,35^ All studies, except one,^25^ involved adult populations. Most trials employed daily doses of lurasidone ranging from 20 to 120 mg, either fixed doses^26,28,31–34^ or flexibly dosed regimes.^14,24,25,27,29,35,36^ One study^30^ specifically re-randomised early non-responders to lurasidone 80 mg to either lurasidone 80 or 160 mg for the final 4 weeks of the trial. Of the 14 studies, 13 were 6 weeks long while one^24^ was 28 weeks in length and was conducted following a 12- to 20-week initial stabilisation phase with open-label lurasidone (20–80 mg). All trial reports were published in English and were conducted in multiple centres across the USA, Europe, South America and Asia, in out-patient or in-patient settings.

**Table 1:** Study characteristics

Reference no.	Study design	Population^a^	Diagnosis^b^	Intervention	Lurasidone dose	Comparator	Follow-up	Primary outcome measure	Country/continent	Setting
24	RCT	496 adults	Bipolar I disorder	Lurasidone (add-on)	20–80 mg flexibly dosed (after stabilisation in OL trial)	Lithium or sodium valproate	28 weeks	MADRS	USA, Europe, Asia, South America	Not specified
25	RCT	350 children (10–17 years)	Bipolar I disorder (current MDD episode)	Lurasidone (monotherapy)	20–80 mg flexibly dosed (with 20 mg initiation)	Placebo	6 weeks	CDRS-R	USA, Europe, Asia, South America	In-patient; out-patient
26	RCT	483 adults	Schizophrenia (acute exacerbation)	Lurasidone (monotherapy)	40 mg	Placebo	6 weeks	CDSS	Europe, Asia	In-patient
27	RCT	525 adults	Bipolar I disorder (current MDD episode)	Lurasidone (monotherapy)	20–60 or 80–120 mg flexibly dosed	Placebo	6 weeks	MADRS	Europe, Asia	Out-patient
28	RCT	368 adults	Schizophrenia (recent acute exacerbation)	Lurasidone (monotherapy)	80 or 160 mg	Placebo	6 weeks	MADRS	USA, Europe, Asia, South America	In-patient
29	RCT	348 adults	Bipolar I disorder (current MDD episode)	Lurasidone (add-on)	20–120 mg flexibly dosed	Lithium or sodium valproate	6 weeks	MADRS	Europe, Asia, Africa, North America	Out-patient
29	RCT	505 adults	Bipolar I disorder (current MDD episode)	Lurasidone (monotherapy)	20–60 or 80–120 mg flexibly dosed	Placebo	6 weeks	MADRS	USA, Europe, Asia, South Africa	Out-patient
30	RCT	412 adults	Schizophrenia (acute exacerbation)	Lurasidone (monotherapy)	20 or 80–160 mg (dosed depending on early response)	Placebo	6 weeks	MADRS	USA, Europe, Asia, South America	In-patient
31	RCT	355 adults	Schizophrenia (acute exacerbation)	Lurasidone (monotherapy)	40 or 120 mg	Placebo	6 weeks	MADRS	USA, Europe, Asia, South America	In-patient
32	RCT	180 adults	Schizophrenia (acute exacerbation)	Lurasidone (monotherapy)	80 mg	Placebo	6 weeks	MADRS	USA	In-patient
33	RCT	500 adults	Schizophrenia (acute exacerbation)	Lurasidone (monotherapy)	40, 80 or 120 mg	Placebo	6 weeks	MADRS	USA, Europe, Asia	In-patient
34	RCT	284 adults	Schizophrenia (acute exacerbation)	Lurasidone (monotherapy)	20, 40 or 80 mg	Placebo	6 weeks	MADRS	USA	In-patient
35	RCT	356 adults	Bipolar I disorder (current MDD episode)	Lurasidone (add-on)	20–120 mg flexibly dosed	Lithium or sodium valproate	6 weeks	MADRS	Europe, Asia, South and North America	Out-patient
35	RCT	211 adults	MDD with mixed features	Lurasidone (monotherapy)	20–60 mg flexibly dosed	Placebo	6 weeks	MADRS	Europe, USA	Out-patient

CDRS-R, Children’s Depression Rating Scale – Revised; CDSS, Calgary Depression Scale for Schizophrenia; MADRS, Montgomery–Asberg Depression Rating Scale; MDD, major depressive disorder; OL, open label; RCT, randomised controlled trial.

a Randomised population.

b Utilising DSM-4 or DSM-5-TR criteria.

### Risk of bias assessment

Quality assessment data are displayed in the risk of bias tables, including explanatory notes and overall bias across trials (Supplementary materials 9A). In total, nine trials were assessed as having a low risk of bias^14,24–27,29,31,35,36^ and the five remaining trials had a high risk of bias.^28,30,32–34^ All studies carried out an intention-to-treat analysis, and had outcome assessors masked to treatment allocation. The main reason for downgrading several trials was a failure to perform appropriate efficacy analyses that would have accounted for the missing data arising from these studies’ high withdrawal rates (Supplementary materials 9B, C).

### Data analysis

**Fig. 2:**
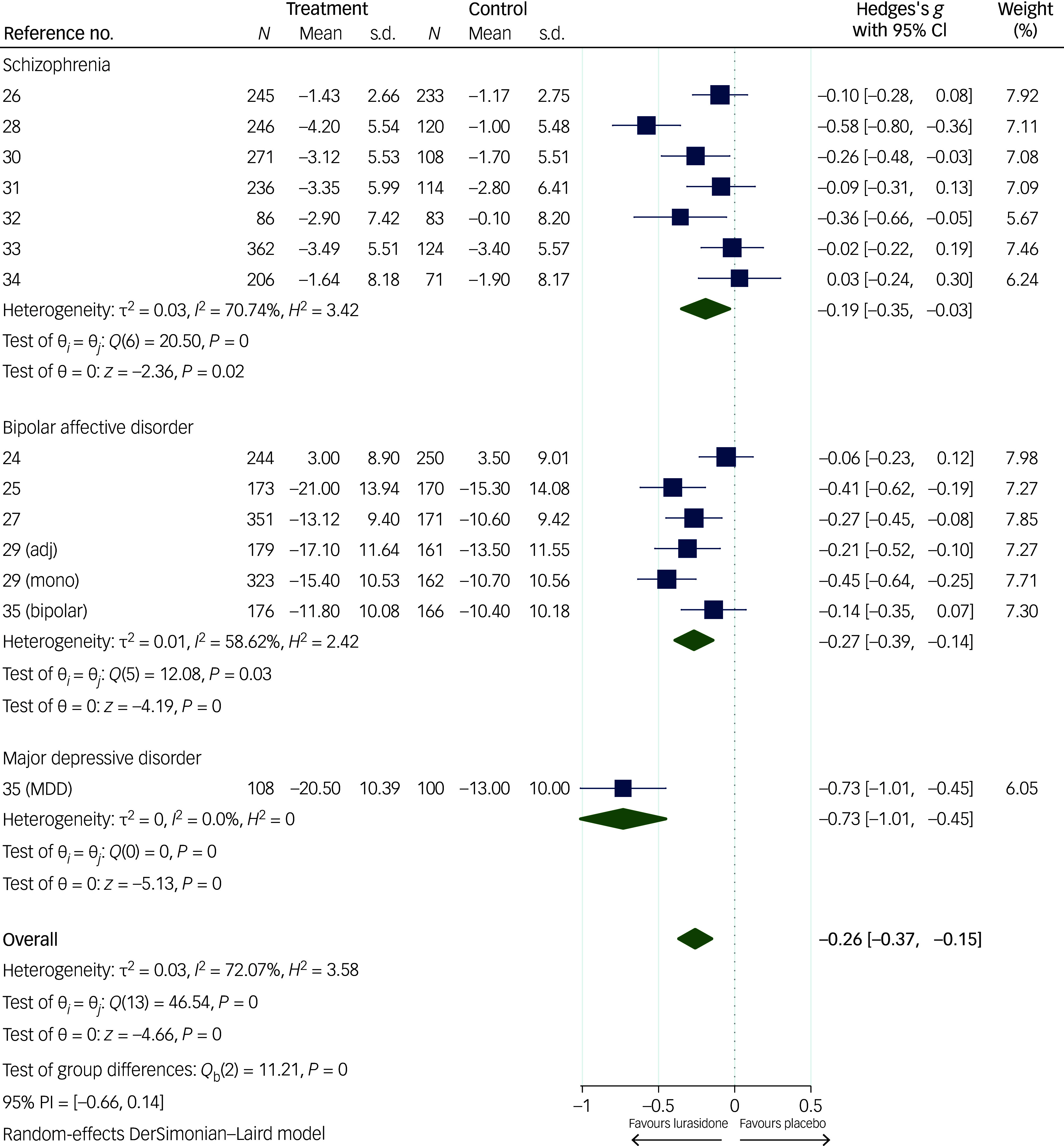
Forest plot of efficacy meta-analysis (mean value for depressive symptoms at end-point). adj, adjunctive therapy; MDD, major depressive disorder; mono, monotherapy; PI, prediction interval.

**Fig. 3:**
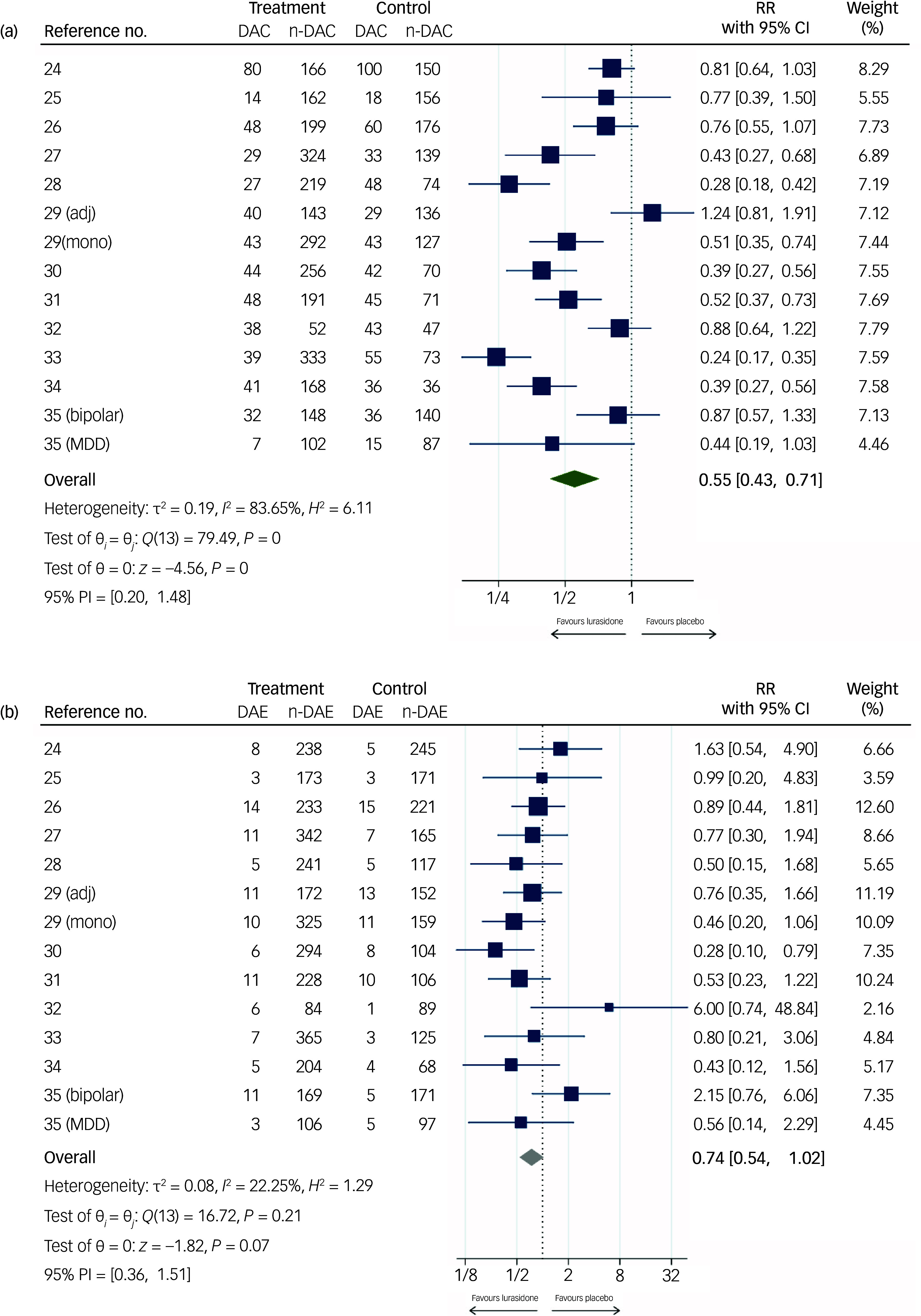
Forest plots for (a) acceptability (number of participants discontinuing treatment due to any cause) and (b) tolerability (numbers of participants discontinuing treatment due to adverse events). RR, relative risk; DAC, discontinuation due to any cause; n-DAC, non-discontinuation due to any cause; DAE, discontinuation due to adverse events; n-DAE, non-discontinuation due to adverse events; adj, adjunctive therapy; MDD, major depressive disorder; mono, monotherapy; PI, prediction interval.

The forest plots in Fig. 2 and Fig. 3 display the effect sizes for all outcomes (SMD for continuous outcomes, relative risk for dichotomous outcomes) with 95% CI from each trial, as well as pooled results and heterogeneity. Certainty of the evidence is reported in the GRADE table (Supplementary materials 10).

#### Efficacy

Fourteen trials comprising 5239 participants were included in the meta-analysis for the primary outcome of efficacy as mean value for depressive symptoms (on any standardised scale) at study end-point (Fig. 2). This showed a statistically significant improvement in favour of lurasidone with small effect size (SMD −0.26, 95% CI −0.37, −0.15). This outcome was scored as having a ‘low’ degree of certainty due to serious concerns about risk of bias and inconsistency (Supplementary materials 10). No publication bias was identified (Supplementary materials 8A–D).

There was substantial heterogeneity (*I* ^2^ = 72.07%), which seemed to be mainly driven by one study.^36^ This was the only trial we identified conducted in people diagnosed with MDD, and it found a larger benefit of lurasidone (*n* = 208; Hedge’s *g* −0.73, 95% CI −1.01, −0.45) compared with all other trials. However, in a sensitivity analysis excluding this trial (Supplementary materials 5A), results remained consistent with the primary analysis (SMD −0.23, 95% CI −0.33, −0.13), with no significant change for between-studies variability, which remained substantial (*I* ^2^ = 65.13%).

Further sensitivity analyses (Supplementary materials 5B−F) did not materially change the benefit on depressive symptoms seen for lurasidone treatment compared with placebo, regardless of the exclusion of trials where lurasidone was used as add-on therapy,^24,29,35^ the one trial with longer follow-up,^24^ trials in younger populations^25^ and trials scored as being at high risk of bias.^28,30,32–34^ Three trials^24,29,35^ used lurasidone as an adjunctive therapy, and there was weaker evidence of a beneficial effect on depression scores in the sensitivity analysis including only these studies (SMD −0.35, 95% CI −0.71, 0.01).

A subgroup analysis (Supplementary materials 6A) of trials where lurasidone was used in people with bipolar disorder^14,24,25,27,29,35^ is in line with the primary analysis (SMD −0.27, 95% CI −0.39, −0.14), while the subgroup analysis of trials in schizophrenia^26,28,30–34^ (Supplementary materials 6B) provided weaker evidence of treatment benefit (SMD −0.19, 95% CI −0.35, −0.03).

#### Acceptability and tolerability

Treatment acceptability (Fig. 3(a)) was slightly better for the lurasidone group (*n* = 5370; relative risk 0.55, 95% CI 0.43, 0.71; low certainty), with a high degree of heterogeneity (*I* ^2^ = 83.65%).

Treatment tolerability (Fig. 3(b)) was slightly worse in the lurasidone compared with the control group (*n* = 5370; relative risk 0.74, 95% CI 0.54, 1.02; moderate certainty), with low heterogeneity (*I* ^2^ = 22.25%). However, this was not statistically significant.

#### Safety

All trials consistently reported the number of patients experiencing any adverse events, and pooled estimates showed better safety outcomes for lurasidone (*n* = 5370; relative risk 0.73, 95% CI 0.58, 0.91; low certainty) compared with placebo (Supplementary materials 7). The most commonly reported side effects were nausea, headache, akathisia, sedation and insomnia (Supplementary materials 13B).

## Discussion

This systematic review and meta-analysis of randomised controlled studies supports the efficacy of lurasidone in reducing the severity of depressive symptoms in people with different mental health diagnoses (bipolar depression, schizophrenia and MDD with mixed features), with an overall small effect size. The main strength of our study is the use of a transdiagnostic approach^7^ in exploring the benefits of this atypical antipsychotic for depressive symptoms, which complicate recovery and increase morbidity across mental health conditions. We utilised a broad strategy in our database search to capture trials conducted in patients with any psychiatric diagnosis.

The transdiagnostic approach is increasingly recognised as a valuable framework in psychiatry, with ongoing systematic initiatives aiming to formalise its implementation in both research and clinical settings.^37,38^ Dimensional models such as the Hierarchical Taxonomy of Psychopathology (HiTOP)^38^ offer a conceptual framework for using psychotherapeutic and psychopharmacological interventions that target underlying symptom domains present across multiple disorders. This mirrors clinical practice, where medication choice is often guided by presenting symptom clusters rather than by strict diagnostic labels (e.g. antidepressants and antipsychotics, which are frequently prescribed across disorders to address overlapping symptoms such as low mood, irritability or sleep disturbance). To date, transdiagnostic systematic reviews in psychiatry have focused on psychotherapeutic interventions,^39,40^ and there appears to be less published research evaluating pharmacological interventions using this framework.^41^ It is therefore challenging to directly compare our results with those of previous meta-analyses. As such, we considered the individual included diagnoses separately utilising our subgroup findings; these results are consistent with previous meta-analyses demonstrating a small to moderate benefit of lurasidone on depression symptoms within specific diagnostic domains. A recent NMA in bipolar depression^18^ found a similar beneficial effect size for lurasidone on depressive scores compared with placebo (SMD 0.29, 95% CI 0.14, 0.45), which is in agreement with our bipolar disorder subgroup analysis (Supplementary materials 6A). In this network meta-analysis,^18^ lurasidone ranked fourth for reducing bipolar depression symptoms after quetiapine + fluoxetine (SMD 0.41, 95% CI 0.19, 0.64), quetiapine monotherapy (SMD 0.35, 95% CI 0.23, 0.47) and olanzapine (SMD 0.35, 95% CI 0.15, 0.54). Interestingly, our sensitivity analysis of the three included trials^24,29,35^ using lurasidone as add-on to lithium or sodium valproate, rather than as monotherapy, showed weaker evidence for efficacy on depression symptoms (SMD −0.35, 95% CI −0.71, 0; Supplementary materials 5C), indicating a need for further trials to clarify the added benefits of lurasidone in patients already established on mood stabilisers.

Our findings related to the efficacy of lurasidone in managing depressive symptoms in schizophrenia are less conclusive. A subgroup analysis of studies in schizophrenia (Supplementary materials 6B) showed weaker evidence for a small beneficial effect (SMD −0.19, 95% CI −0.35, −0.03), with high heterogeneity of results. This is consistent with previous NMA data^19^ showing reduction in depressive symptoms with lurasidone in schizophrenia (SMD −0.20, 95% CI −0.32, −0.09), but several antipsychotics performed more favourably with greater confidence in the evidence, including amiloride (SMD −0.44, 95% CI −0.60, −0.28) and olanzapine (SMD −0.37, 95% CI −0.46, −0.29). One explanation of our findings in schizophrenia could be the use of flexible dosing regimens or various lurasidone doses in the different arms of the included trials, which we combined into a single intervention group for the pairwise analysis. Indeed, an NMA of dose–response effects of lurasidone in acute schizophrenia^42^ found that higher doses had larger therapeutic benefit – the half-maximal effect doses for Positive and Negative Syndrome Scale total (ED_50_ 160 mg/day) and MADRS (ED_50_ 103.68 mg/day) score reduction were higher than 80 mg/day. Similarly, a recent meta-analysis^43^ found no effects of lurasidone 40 mg (SMD −1.53) and 120 mg (SMD 0.17) in acute schizophrenia, but 80 mg (SMD −2.90) and 160 mg (SMD −6.78) regimens significantly reduced depression scores, suggesting a dose dependence in lurasidone’s efficacy. It is also worth noting the complex overlap between depressive and negative symptoms, including anhedonia and avolition, in people with schizophrenia.^44^ Future trials in schizophrenia spectrum disorders should consider higher lurasidone doses to achieve an antidepressant effect, and might explore how this compares with reduction in negative symptoms scales.

The study conducted in patients with MDD with subthreshold hypomanic features^36^ showed the largest effect size in sensitivity analyses. This diagnostic entity, newly defined in DSM-5,^45^ represents an intermediate phenotype between MDD and bipolar disorder for which standard antidepressants may not have adequate efficacy.^46^ In a previous meta-analysis of antipsychotic monotherapy in MDD,^20^ response rates with lurasidone based on the same trial that we identified^36^ (relative risk 2.14, 95% CI 1.55, 2.96) were comparable to those for amisulpride (relative risk 2.17, 95% CI 1.42, 3.30) and higher than for both olanzapine (relative risk 1.18, 95% CI 0.79, 1.76) and quetiapine (relative risk 1.47, 95% CI 1.21, 1.79), although no head-to-head comparison was performed. While antipsychotics as either monotherapy or add-ons for unipolar depression have previously been shown to be efficacious,^20^ lurasidone may be uniquely suited to targeting atypical forms of depression through its multimodal pharmacological profile.^17^ In addition to its antagonism at D2 dopamine receptors, lurasidone has high affinity for 5-HT7 and partial agonist activity at 5-HT1A receptors,^17^ and there is evidence supporting altered function of these receptors across psychiatric illnesses.^47,48^ Modulation of 5HT1A function has been hypothesised to contribute to the antidepressant effects of both traditional antidepressants and atypical antipsychotics.^47^ Similarly, the 5HT7 receptor has been implicated in animal models of depression and schizophrenia,^48^ and lurasidone’s antidepressant effects are absent in 5-HT7 knockout mice.^49^ Other putative antidepressant mechanisms of lurasidone include increased neuroplasticity in the prefrontal cortex^50^ and increased serotonergic transmission in the dorsal raphe nucleus secondary to desensitisation of 5-HT1A and 5-HT7 receptors.^51^ Preclinical evidence therefore supports the findings of this meta-analysis, suggesting the potential of lurasidone as a transdiagnostic antidepressant.

Analysis of our secondary outcomes showed that both acceptability and safety were significantly better for the lurasidone group, with no statistically significant difference for tolerability. Commonly reported side-effects for lurasidone across studies were nausea, headache, akathisia, sedation and insomnia. Previous studies have shown lurasidone to have a more favourable metabolic risk profile compared with many other antipsychotics, while being associated with a smaller risk of extrapyramidal effects and prolactin elevation.^19^ Although, to our knowledge, there are no studies directly comparing the safety of lurasidone with that of other psychotropics, such as antidepressants, adoption of a transdiagnostic approach could encourage the comparison of psychopharmacological classes traditionally thought of as discrete, and facilitate personalised treatment decisions.

Our search also identified two further eligible RCTs of lurasidone in bipolar disorder, with results yet to be published.^52,53^ One of these (ELICE-BD) is still ongoing and is planned to assess the cognitive effects of lurasidone compared with placebo in euthymic bipolar patients, in addition to its effects on depressive symptom severity. Given the putative pro-cognitive effects of lurasidone suggested by its affinity profile,^49^ and preliminary clinical evidence supporting this,^54,55^ future RCTs such as ELICE-BD might shed light on lurasidone’s potential as a transdiagnostic strategy for tackling not only problematic depressive symptoms, but also cognitive deficits commonly encountered across diagnostic domains. As discussed above, the involvement of overlapping pathophysiological and neurobiological pathways across psychiatric disorders supports a transdiagnostic approach to the evaluation of pharmacotherapies. We expect this model to be applicable not only to lurasidone, but also to other psychopharmacological agents that target shared underlying mechanisms and symptom profiles. Future validation of this approach should follow emerging standards, such as the TRANSD recommendations,^7,38^ which offer methodological guidance for improving the design, reporting and interpretation of transdiagnostic research.

### Limitations

The present study has several limitations, including the high risk of bias of several included studies and the high heterogeneity of our findings, both of which have contributed to a low certainty of evidence, as well as the relatively short duration of follow-up.

The high RoB2 was primarily driven by several trials improperly handling missing data, mostly through the use of an unsuitable method such as simple imputation through ‘last observation carried forward’, which assumes that data were missing completely at random. Missing outcome data is not uncommon in mental health trials,^56^ yet it is handled inconsistently across the literature. Some included trials did use more appropriate methods (such as mixed models for repeated measures) to account for missing data for their primary outcome, which depressive symptoms were not always defined as. When considering research methodology utilising a transdiagnostic framework, the current prototypical RCT design may be ill-suited.^6^ Novel approaches – for example, defining multiple co-primary outcomes – would better lend themselves to investigating therapeutic agents with multimodal pharmacological effects, such as lurasidone. Nevertheless, a subgroup analysis excluding the five studies with high RoB2 did not alter our results.

While we found evidence of significant overall efficacy of lurasidone on depressive symptoms, we also identified substantial heterogeneity among the studies included. Although we carried out several supplementary analyses, this did not materially reduce heterogeneity, which could reflect an inherent limitation of assessing interventions transdiagnostically, because pooling trials conducted in different psychiatric diagnoses is bound to introduce a degree of clinical heterogeneity. Furthermore, the RCTs included were relatively large and were conducted across multiple countries, centres and clinical settings. Although these also utilised different dosing regimens of lurasidone, we did not perform a dose–effect meta-analysis in this review. Future research should consider variables related to dosing, acuity of presentation and healthcare context.

All trials included disclosed sponsorship by the pharmaceutical company that manufactures lurasidone, and several were authored by employees of that company. Another limitation of the available evidence involves the short follow-up periods, with study end-point at 6 weeks, and therefore we cannot exclude the possibility that more robust antidepressant effects might occur over longer follow-up periods. However, several trials^14,25,27,29,35,36^ were followed by longer-term, open-label extension studies. In summary, these found maintained improvements in depressive symptoms and favourable safety parameters with lurasidone from 3 months to 2 years in patients with MDD or bipolar depression (Supplementary materials 12). A formal review of these continuation studies, and more long-term, high-quality RCTs, would shed light on the longer-term efficacy and safety profile of lurasidone, particularly in clinically stable subjects outside of acute exacerbations.

Finally, we presented the magnitude of effect sizes as per Cochrane guidance, which is the norm in the field. While it would have aided interpretation, we did not conduct a responder analysis in this study^57^ because response rates for depression scores were not reported in many of the included trials, and we did not impute these from continuous outcomes to avoid introducing bias.^58^

Overall, our findings suggest that lurasidone is a useful psychopharmacological tool for treating depressive symptoms in a variety of patients with severe mental illness, supporting the current evidence base which has, to date, guided clinical recommendations based on expert opinion. To support updates to clinical guidelines, further large-scale trials with robust designs, longer follow-up and pragmatic dosing regimens are needed. Future research examining depressive symptoms transdiagnostically may also identify patient subgroups who would most benefit from lurasidone, promoting precision psychiatry approaches and enabling tailored therapeutic decisions beyond diagnostic boundaries.

## Supporting information

Ghenciulescu et al. supplementary material Ghenciulescu et al. supplementary material

## Data Availability

The authors confirm that the data supporting the findings of the study are available within the article and its supplementary materials.

## Supplementary material

The supplementary material is available online at https://doi.org/10.1192/bjp.2025.10343
