# Supplementary material for: Transdiagnostic efficacy of lurasidone on depressive symptoms: systematic review and meta-analysis of randomised controlled trials
Source: Br J Psychiatry. 2025 Aug 20;228(4):357–66. doi: 10.1192/bjp.2025.10343 (PMC13051213; doi:10.1192/bjp.2025.10343)
Supplement: Ghenciulescu et al. supplementary material [file S0007125025103437sup001.docx]

**Supplementary Materials**

**Supplementary material S1A. PRISMA 2020 Checklist**

| **Section and Topic** | **Item #** | **Checklist item** | **Location where item is reported** |
| --- | --- | --- | --- |
| **TITLE** | | |  |
| Title | 1 | Identify the report as a systematic review. | Page 4 |
| **ABSTRACT** | | |  |
| Abstract | 2 | See the PRISMA 2020 for Abstracts checklist. | Supplementary material S1B |
| **INTRODUCTION** | | |  |
| Rationale | 3 | Describe the rationale for the review in the context of existing knowledge. | Pages 6-7 |
| Objectives | 4 | Provide an explicit statement of the objective(s) or question(s) the review addresses. | Page 7 |
| **METHODS** | | |  |
| Eligibility criteria | 5 | Specify the inclusion and exclusion criteria for the review and how studies were grouped for the syntheses. | Pages 8-10 |
| Information sources | 6 | Specify all databases, registers, websites, organisations, reference lists and other sources searched or consulted to identify studies. Specify the date when each source was last searched or consulted. | Page 8 |
| Search strategy | 7 | Present the full search strategies for all databases, registers and websites, including any filters and limits used. | Supplementary material S2 |
| Selection process | 8 | Specify the methods used to decide whether a study met the inclusion criteria of the review, including how many reviewers screened each record and each report retrieved, whether they worked independently, and if applicable, details of automation tools used in the process. | Pages 9, 10 |
| Data collection process | 9 | Specify the methods used to collect data from reports, including how many reviewers collected data from each report, whether they worked independently, any processes for obtaining or confirming data from study investigators, and if applicable, details of automation tools used in the process. | Pages 9, 10 |
| Data items | 10a | List and define all outcomes for which data were sought. Specify whether all results that were compatible with each outcome domain in each study were sought (e.g. for all measures, time points, analyses), and if not, the methods used to decide which results to collect. | Page 9 |
|  | 10b | List and define all other variables for which data were sought (e.g. participant and intervention characteristics, funding sources). Describe any assumptions made about any missing or unclear information. | Page 9 |
| Study risk of bias assessment | 11 | Specify the methods used to assess risk of bias in the included studies, including details of the tool(s) used, how many reviewers assessed each study and whether they worked independently, and if applicable, details of automation tools used in the process. | Page 10 |
| Effect measures | 12 | Specify for each outcome the effect measure(s) (e.g. risk ratio, mean difference) used in the synthesis or presentation of results. | Page 10 |
| Synthesis methods | 13a | Describe the processes used to decide which studies were eligible for each synthesis (e.g. tabulating the study intervention characteristics and comparing against the planned groups for each synthesis (item #5)). | / |
|  | 13b | Describe any methods required to prepare the data for presentation or synthesis, such as handling of missing summary statistics, or data conversions. | Page 10 |
|  | 13c | Describe any methods used to tabulate or visually display results of individual studies and syntheses. | Page 10 |
|  | 13d | Describe any methods used to synthesize results and provide a rationale for the choice(s). If meta-analysis was performed, describe the model(s), method(s) to identify the presence and extent of statistical heterogeneity, and software package(s) used. | Page 10 |
|  | 13e | Describe any methods used to explore possible causes of heterogeneity among study results (e.g. subgroup analysis, meta-regression). | Page 10 |
|  | 13f | Describe any sensitivity analyses conducted to assess robustness of the synthesized results. | Page 10 |
| Reporting bias assessment | 14 | Describe any methods used to assess risk of bias due to missing results in a synthesis (arising from reporting biases). | Page 10 |
| Certainty assessment | 15 | Describe any methods used to assess certainty (or confidence) in the body of evidence for an outcome. | Page 10 |
| **RESULTS** | | |  |
| Study selection | 16a | Describe the results of the search and selection process, from the number of records identified in the search to the number of studies included in the review, ideally using a flow diagram. | Page 11 |
|  | 16b | Cite studies that might appear to meet the inclusion criteria, but which were excluded, and explain why they were excluded. | / |
| Study characteristics | 17 | Cite each included study and present its characteristics. | Page 11, Table 1 |
| Risk of bias in studies | 18 | Present assessments of risk of bias for each included study. | Page 16; Supplementary material S4 |
| Results of individual studies | 19 | For all outcomes, present, for each study: (a) summary statistics for each group (where appropriate) and (b) an effect estimate and its precision (e.g. confidence/credible interval), ideally using structured tables or plots. | Supplementary material S3 |
| Results of syntheses | 20a | For each synthesis, briefly summarise the characteristics and risk of bias among contributing studies. | Pages 11, 16 |
|  | 20b | Present results of all statistical syntheses conducted. If meta-analysis was done, present for each the summary estimate and its precision (e.g. confidence/credible interval) and measures of statistical heterogeneity. If comparing groups, describe the direction of the effect. | Pages 16-18, Figure 2, Figure 3 |
|  | 20c | Present results of all investigations of possible causes of heterogeneity among study results. | Page 24, Supplementary Materials S6-8 |
|  | 20d | Present results of all sensitivity analyses conducted to assess the robustness of the synthesized results. | Supplementary Material S7 |
| Reporting biases | 21 | Present assessments of risk of bias due to missing results (arising from reporting biases) for each synthesis assessed. | S6 |
| Certainty of evidence | 22 | Present assessments of certainty (or confidence) in the body of evidence for each outcome assessed. | Supplementary Material S5 |

| **DISCUSSION** | | |  |
| --- | --- | --- | --- |
| Discussion | 23a | Provide a general interpretation of the results in the context of other evidence. | Pages 21-23 |
|  | 23b | Discuss any limitations of the evidence included in the review. | Pages 23-24 |
|  | 23c | Discuss any limitations of the review processes used. | Pages 23-24 |
|  | 23d | Discuss implications of the results for practice, policy, and future research. | Pages 24-25 |
| **OTHER INFORMATION** | | |  |
| Registration and protocol | 24a | Provide registration information for the review, including register name and registration number, or state that the review was not registered. | Page 8 |
|  | 24b | Indicate where the review protocol can be accessed, or state that a protocol was not prepared. | Page 8, Abstract |
|  | 24c | Describe and explain any amendments to information provided at registration or in the protocol. | Supplementary Material S10 |
| Support | 25 | Describe sources of financial or non-financial support for the review, and the role of the funders or sponsors in the review. | Page 4 |
| Competing interests | 26 | Declare any competing interests of review authors. | Page 4 |
| Availability of data, code and other materials | 27 | Report which of the following are publicly available and where they can be found: template data collection forms; data extracted from included studies; data used for all analyses; analytic code; any other materials used in the review. | Supplementary Material S3 |

*From:* Page MJ, McKenzie JE, Bossuyt PM, Boutron I, Hoffmann TC, Mulrow CD, et al. The PRISMA 2020 statement: an updated guideline for reporting systematic reviews. BMJ 2021;372:n71. doi: 10.1136/bmj.n71

**Supplementary material S1B. PRISMA 2020 for Abstract Checklist**

| **Section and Topic** | **Item #** | **Checklist item** | **Reported (Yes/No)** |
| --- | --- | --- | --- |
| **TITLE** | | |  |
| Title | 1 | Identify the report as a systematic review. | Yes |
| **BACKGROUND** | | |  |
| Objectives | 2 | Provide an explicit statement of the main objective(s) or question(s) the review addresses. | Yes |
| **METHODS** | | |  |
| Eligibility criteria | 3 | Specify the inclusion and exclusion criteria for the review. | Yes |
| Information sources | 4 | Specify the information sources (e.g. databases, registers) used to identify studies and the date when each was last searched. | Yes |
| Risk of bias | 5 | Specify the methods used to assess risk of bias in the included studies. | Yes |
| Synthesis of results | 6 | Specify the methods used to present and synthesise results. | Yes |
| **RESULTS** | | |  |
| Included studies | 7 | Give the total number of included studies and participants and summarise relevant characteristics of studies. | Yes |
| Synthesis of results | 8 | Present results for main outcomes, preferably indicating the number of included studies and participants for each. If meta-analysis was done, report the summary estimate and confidence/credible interval. If comparing groups, indicate the direction of the effect (i.e. which group is favoured). | Yes |
| **DISCUSSION** | | |  |
| Limitations of evidence | 9 | Provide a brief summary of the limitations of the evidence included in the review (e.g. study risk of bias, inconsistency and imprecision). | Yes |
| Interpretation | 10 | Provide a general interpretation of the results and important implications. | Yes |
| **OTHER** | | |  |
| Funding | 11 | Specify the primary source of funding for the review. | n/a |
| Registration | 12 | Provide the register name and registration number. | Yes |

*From:*  Page MJ, McKenzie JE, Bossuyt PM, Boutron I, Hoffmann TC, Mulrow CD, et al. The PRISMA 2020 statement: an updated guideline for reporting systematic reviews. BMJ 2021;372:n71. doi: 10.1136/bmj.n71

**Supplementary material S2. Search strategy used for various platforms**

**Embase: inception to October 25, 2024**

| **#** | **Query** | **Results from 25 Oct 2024** |
| --- | --- | --- |
| 1 | lurasidone.mp. or exp lurasidone/ | 2,870 |
| 2 | exp Randomized Controlled Trial/ | 853,487 |
| 3 | exp double blind procedure/ | 225,356 |
| 4 | exp crossover procedure/ | 80,187 |
| 5 | exp randomization/ | 100,652 |
| 6 | randomized controlled trial.tw. | 153,940 |
| 7 | (random$ adj5 control$ adj5 trial$).mp. | 1,253,780 |
| 8 | (double$ adj (blind$ or mask$)).mp. | 319,130 |
| 9 | (random$ adj5 (assign$ or allocat$ or assort$ or reciev$ or receiv$)).mp. | 355,268 |
| 10 | (random$ adj5 control$ adj5 clinic$ adj5 trial$).mp. | 53,000 |
| 11 | Double-Blind.mp. | 308,803 |
| 12 | (clin$ adj2 trial).mp. | 1,901,362 |
| 13 | crossover.mp. | 124,287 |
| 14 | 2 or 3 or 4 or 5 or 6 or 7 or 8 or 9 or 10 or 11 or 12 or 13 | 2,743,736 |
| 15 | 1 and 14 | 1,136 |

**Medline** (Ovid MEDLINE® Epub Ahead of Print, In-Process & Other Non-Indexed Citations, Ovid MEDLINE® Daily and Ovid MEDLINE®): **inception to October 25, 2024**

| **#** | **Query** | **Results from 25 Oct 2024** |
| --- | --- | --- |
| 1 | lurasidone.mp. or exp Lurasidone Hydrochloride/ | 760 |
| 2 | exp Randomized Controlled Trial/ | 626,300 |
| 3 | exp Double-Blind Method/ | 181,078 |
| 4 | randomized controlled trial.pt. | 624,643 |
| 5 | (random$ adj5 control$ adj5 trial$).mp. | 948,476 |
| 6 | (double$ adj (blind$ or mask$)).mp. | 236,890 |
| 7 | (random$ adj5 (assign$ or allocat$ or assort$ or reciev$ or receiv$)).mp. | 358,459 |
| 8 | (random$ adj5 control$ adj5 clinic$ adj5 trial$).mp. | 39,891 |
| 9 | Double-Blind.mp. | 228,488 |
| 10 | randomi$.mp. | 1,172,921 |
| 11 | (clinic$ adj2 trial).mp. | 848,314 |
| 12 | controlled clinical trial.pt. | 95,633 |
| 13 | clinical trial.pt. | 540,639 |
| 14 | exp Clinical Trial/ | 1,007,781 |
| 15 | exp cross-over studies/ | 57,764 |
| 16 | exp clinical trial/ | 1,007,781 |
| 17 | (crossover or cross-over).mp. | 116,873 |
| 18 | 2 or 3 or 4 or 5 or 6 or 7 or 8 or 9 or 10 or 11 or 12 or 13 or 14 or 15 or 16 or 17 | 1,800,791 |
| 19 | 1 and 18 | **265** |

**PsycINFO: inception to October 25, 2024**

| **#** | **Query** | **Results from 25 Oct 2024** |
| --- | --- | --- |
| 1 | lurasidone.mp. | 346 |
| 2 | exp Randomized Controlled Trials/ or Randomized Controlled Trial.mp. | 26,009 |
| 3 | exp Placebo/ or Double Blind&.mp. | 35,691 |
| 4 | Double-Blind.mp. | 31,829 |
| 5 | (double$ adj (blind$ or mask$)).mp. | 32,591 |
| 6 | (random$ adj5 (assign$ or allocat$ or assort$ or reciev$ or receiv$)).mp. | 65,843 |
| 7 | (random$ adj5 control$ adj5 clinic$ adj5 trial$).mp. | 3,764 |
| 8 | (random$ adj5 control$ adj5 trial$).mp. | 60,649 |
| 9 | crossover.mp. | 8,733 |
| 10 | randomized controlled trial.pt,tw. | 25,085 |
| 11 | 2 or 3 or 4 or 5 or 6 or 7 or 8 or 9 or 10 | 144,723 |
| 12 | 1 and 11 | **147** |

**Cochrane CENTRAL Library: inception to October 25, 2024**

#1 (lurasidone): ti,ab,kw (Word variations have been searched) 550

#2 MeSH descriptor: [Lurasidone Hydrochloride] explode all trees **151**

**ClinicalTrials.gov: inception to October 25, 2024**

| **Terms** | **Search Results*** | **Entire Database**** |
| --- | --- | --- |
| **lurasidone** | 93 studies | **94 studies** |
| Latuda | 49 studies | 49 studies |

other terms Lurasidone (all studies) = 94

**WHO’s International Clinical Trials Registry Platform (ICTRP): inception to October 25, 2024**

#1 Lurasidone (intervention); ALL trials **82 records for 117 trials found**

**ISRCTN: inception to October 25, 2024**

#1 Filter Interventions: Lurasidone **7 trials**

**Supplementary material S3. PICOS Table**

| **Population** | Participants >=12 years old with any mental health diagnosis |
| --- | --- |
|
| **Intervention** | Lurasidone |
| **Comparison** | Placebo |
| **Outcomes** | Main outcome: Depressive symptoms  Additional outcomes: Acceptability of treatment (proportion of treatment discontinuations due to any cause).  Tolerability of treatment (proportion of treatment discontinuations due to adverse events).  Safety of treatment (type of adverse events, proportion of participants experiencing at least one adverse event). |
| **Study design** | Randomised controlled trials |
|

*From:* Higgins JPT Chandler J Cumpston M Li T Page MJ Welch VA (editors) TJ. *Cochrane Handbook for Systematic Reviews of Interventions version 6.5 (updated August 2024)*. Cochrane. 2024. [www.training.cochrane.org/handbook](http://www.training.cochrane.org/handbook)

**Supplementary material S4. List of all included papers**

Calabrese JR, Pikalov A, Streicher C, Cucchiaro J, Mao Y, Loebel A. Lurasidone in combination with lithium or valproate for the maintenance treatment of bipolar I disorder. *European neuropsychopharmacology : the journal of the European College of Neuropsychopharmacology*. 2017;27(9): 865–876. <https://doi.org/10.1016/J.EURONEURO.2017.06.013>.

DelBello MP, Goldman R, Phillips D, Deng L, Cucchiaro J, Loebel A. Efficacy and Safety of Lurasidone in Children and Adolescents With Bipolar I Depression: A Double-Blind, Placebo-Controlled Study. *Journal of the American Academy of Child and Adolescent Psychiatry*. 2017;56(12): 1015–1025. https://doi.org/10.1016/J.JAAC.2017.10.006.

Iyo M, Ishigooka J, Nakamura M, Sakaguchi R, Okamoto K, Mao Y, et al. Efficacy and safety of lurasidone in acutely psychotic patients with schizophrenia: A 6-week, randomised, double-blind, placebo-controlled study. *Psychiatry and clinical neurosciences*. 2021;75(7): 227–235. https://doi.org/10.1111/PCN.13221.

Kato T, Ishigooka J, Miyajima M, Watabe K, Fujimori T, Masuda T, et al. Double-blind, placebo-controlled study of lurasidone monotherapy for the treatment of bipolar I depression. *Psychiatry and Clinical Neurosciences*. 2020;74(12): 635–644. https://doi.org/10.1111/PCN.13137.

Loebel A, Cucchiaro J, Sarma K, Xu L, Hsu C, Kalali AH, et al. Efficacy and safety of lurasidone 80 mg/day and 160 mg/day in the treatment of schizophrenia: A randomised, double-blind, placebo- and active-controlled trial. *Schizophrenia Research*. 2013;145(1–3): 101–109. https://doi.org/10.1016/J.SCHRES.2013.01.009.

Loebel A, Cucchiaro J, Silva R, Kroger H, Sarma K, Xu J, et al. Lurasidone as adjunctive therapy with lithium or valproate for the treatment of bipolar i depression: A randomised, double-blind, placebo-controlled study. *American Journal of Psychiatry*. 2014;171(2): 169–177. https://doi.org/10.1176/APPI.AJP.2013.13070985/ASSET/IMAGES/LARGE/169F2.JPEG.

Loebel A, Silva R, Goldman R, Watabe K, Cucchiaro J, Citrome L, et al. Lurasidone Dose Escalation in Early Nonresponding Patients With Schizophrenia: A Randomised, Placebo-Controlled Study. *The Journal of Clinical Psychiatry*. 2016;77(12): 10934. https://doi.org/10.4088/JCP.16M10698.

Meltzer HY, Cucchiaro J, Silva R, Ogasa M, Phillips D, Xu J, et al. Lurasidone in the treatment of schizophrenia: A randomised, double-blind, placebo- and olanzapine-controlled study. *American Journal of Psychiatry*. 2011;168(9): 957–967. https://doi.org/10.1176/APPI.AJP.2011.10060907/SUPPL_FILE/AJP_168_09_957_01.PDF.

Nakamura M, Ogasa M, Guarino J, Phillips D, Severs J, Cucchiaro J, et al. Lurasidone in the Treatment of Acute Schizophrenia: A Double-Blind, Placebo-Controlled Trial. *The Journal of Clinical Psychiatry*. 2009;70(6): 10941. https://doi.org/10.4088/JCP.08M04905.

Nasrallah HA, Silva R, Phillips D, Cucchiaro J, Hsu J, Xu J, et al. Lurasidone for the treatment of acutely psychotic patients with schizophrenia: A 6-week, randomised, placebo-controlled study. *Journal of Psychiatric Research*. 2013;47(5): 670–677. https://doi.org/10.1016/J.JPSYCHIRES.2013.01.020.

Potkin SG, Kimura T, Guarino J. A 6-week, double-blind, placebo- and haloperidol-controlled, phase II study of lurasidone in patients with acute schizophrenia. *Therapeutic Advances in Psychopharmacology*. 2015;5(6): 322–331. https://doi.org/10.1177/2045125315606027/ASSET/IMAGES/LARGE/10.1177_2045125315606027-FIG2.JPEG.

Suppes T, Kroger H, Pikalov A, Loebel A. Lurasidone adjunctive with lithium or valproate for bipolar depression: A placebo-controlled trial utilising prospective and retrospective enrolment cohorts. *Journal of Psychiatric Research*. 2016;78: 86–93. https://doi.org/10.1016/J.JPSYCHIRES.2016.03.012.

Suppes T, Silva R, Cucchiaro J, Mao Y, Targum S, Streicher C, et al. Lurasidone for the treatment of major depressive disorder with mixed features: A randomised, double-blind, placebo-controlled study. *American Journal of Psychiatry*. 2016;173(4): 400–407. https://doi.org/10.1176/APPI.AJP.2015.15060770/SUPPL_FILE/APPI.AJP.2015.15060770.DS001.PDF.

**Supplementary material S5. Sensitivity analyses**

**S5A. Sensitivity analysis (heterogeneity – excluding trial in MDD) for efficacy as mean value for depressive symptoms at endpoint**

**
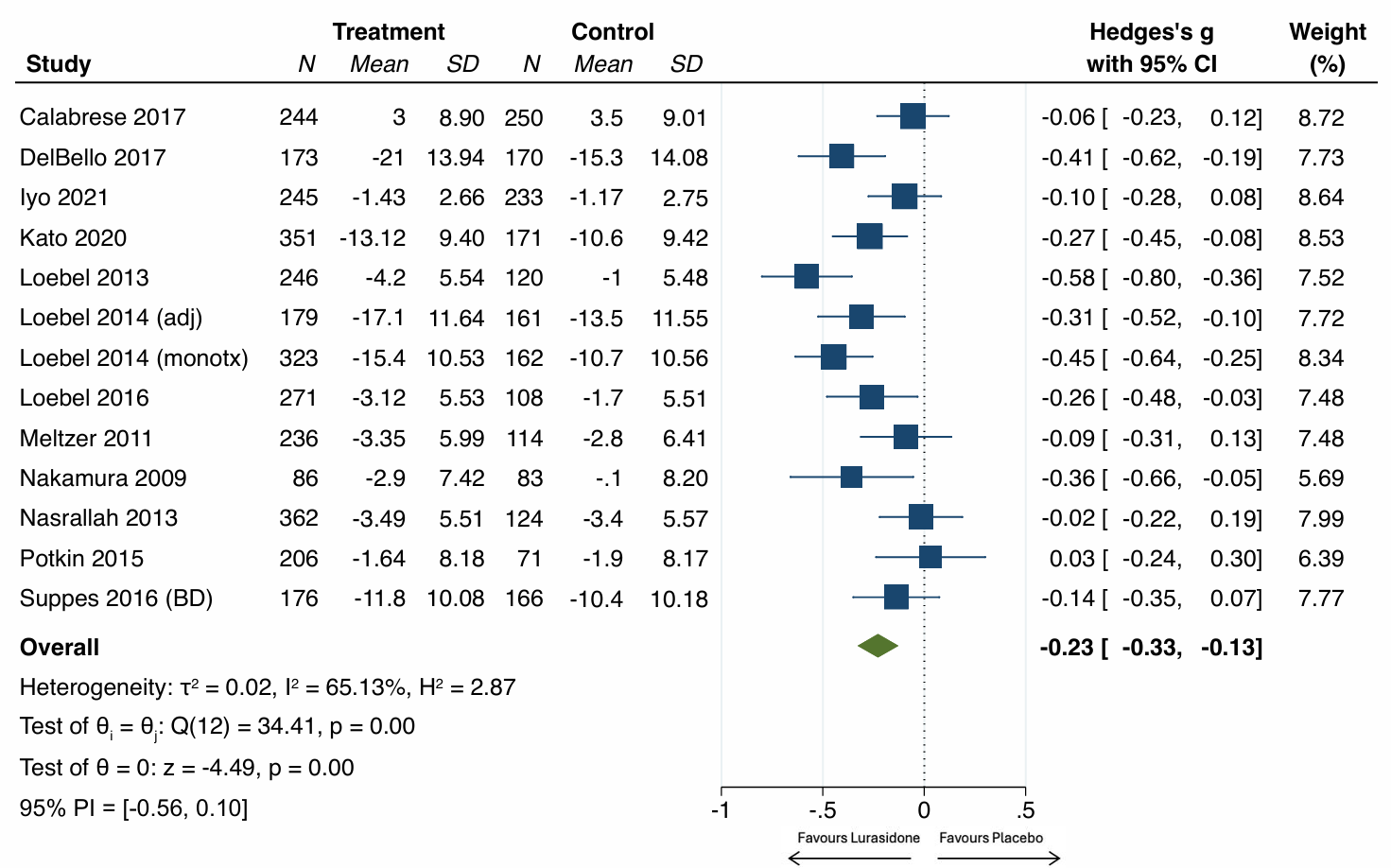
**

**S5B. Sensitivity analysis (only 6-week long trials) for efficacy as mean value for depressive symptoms at endpoint**

**
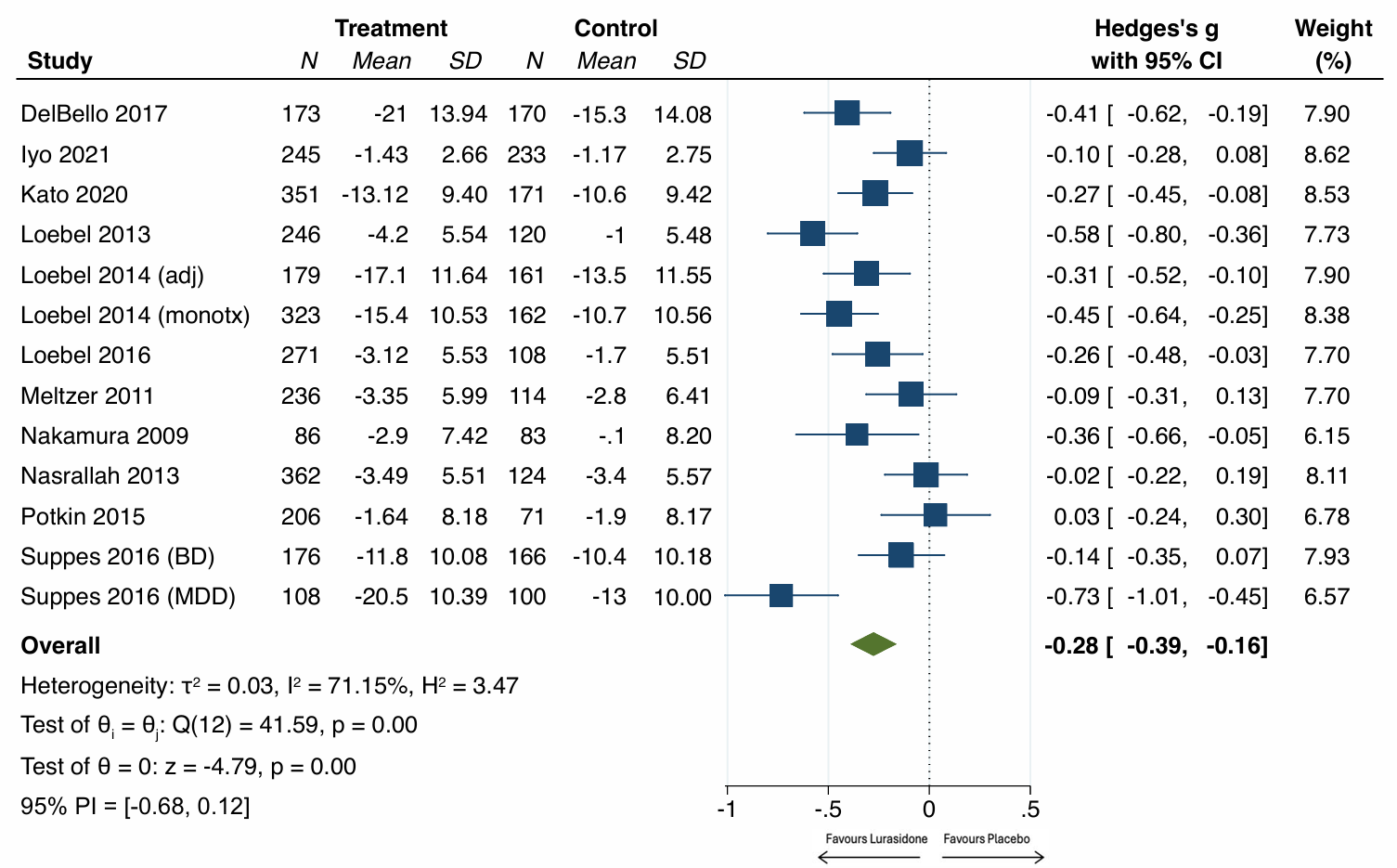
**

**S5C. Sensitivity analysis (only studies with Lurasidone as adjunctive therapy) for efficacy as mean value for depressive symptoms at endpoint**

**
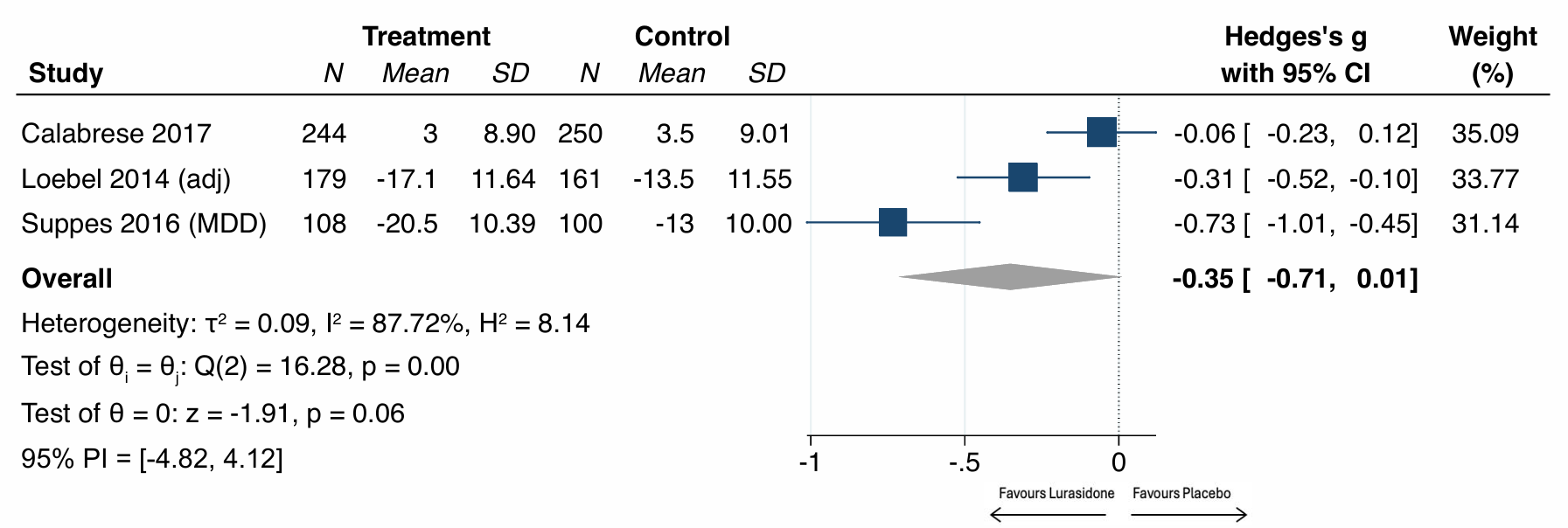
**

**S5D. Sensitivity analysis (only studies with Lurasidone as monotherapy) for efficacy as mean value for depressive symptoms at endpoint**

**
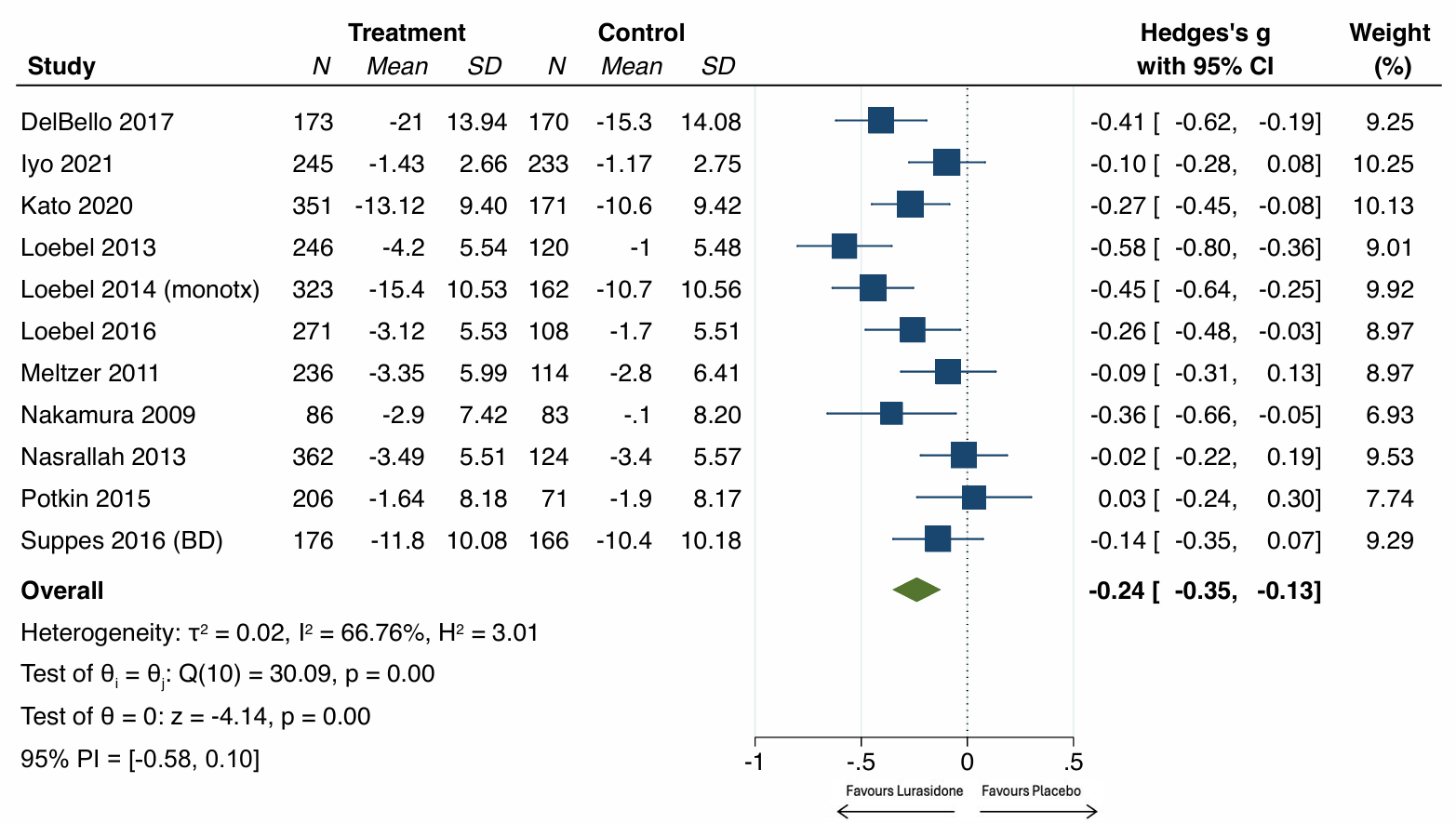
**

**S5E. Sensitivity analysis (only studies in adults) for efficacy as mean value for depressive symptoms at endpoint**

**
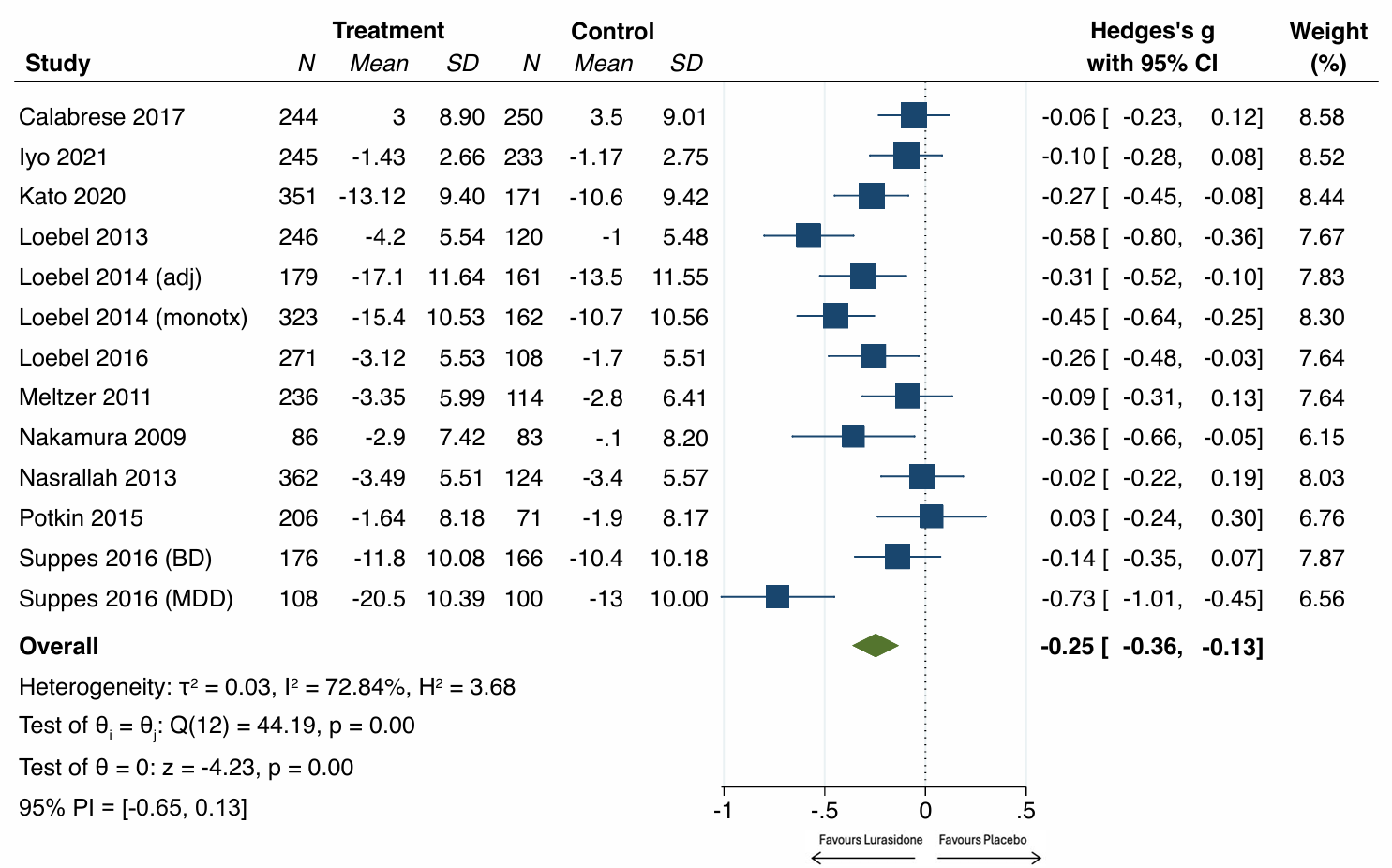
**

**S5F. Sensitivity analysis (excluding studies with high risk of bias) for efficacy as mean value for depressive symptoms at endpoint**

**
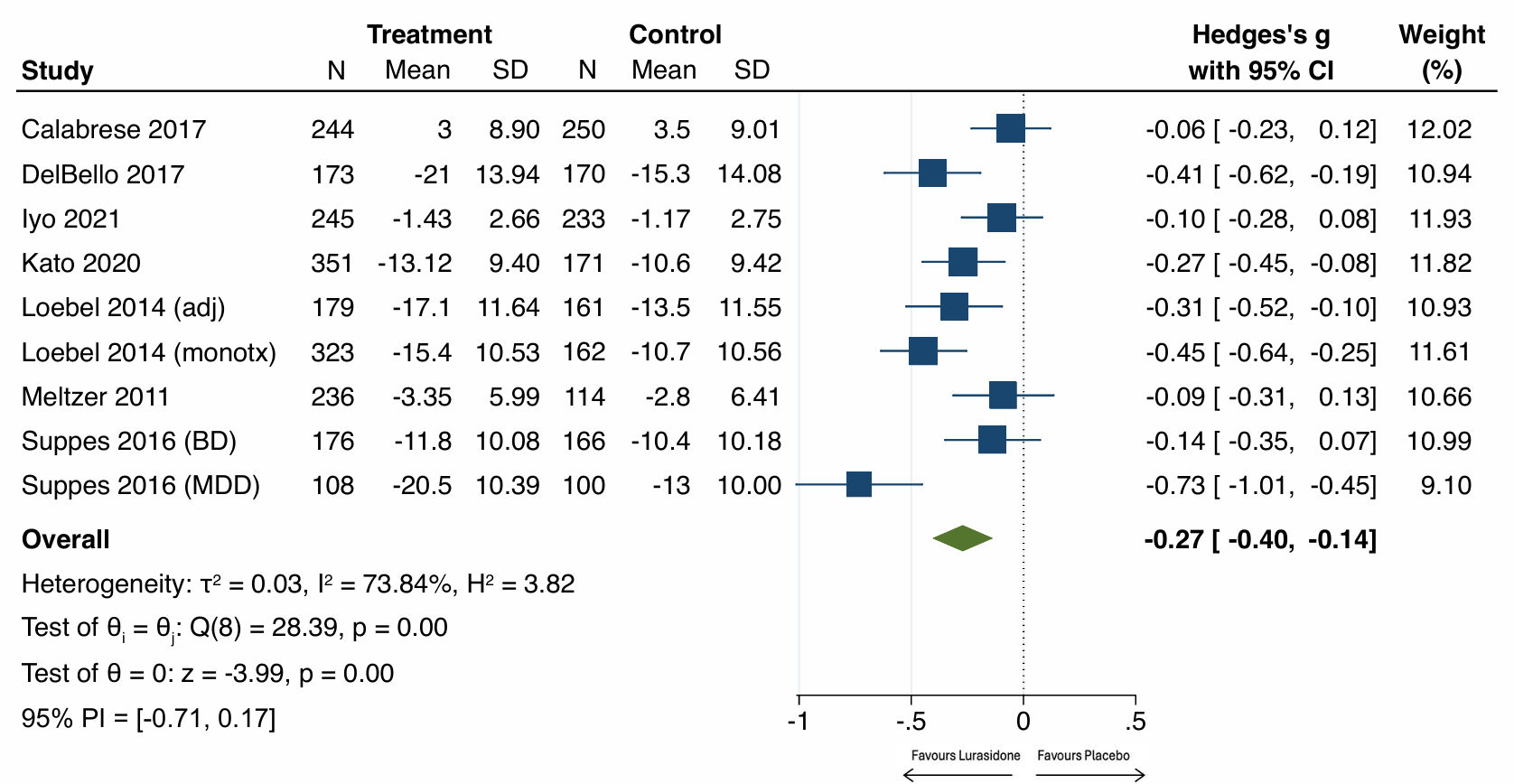
**

**Supplementary material S6. Subgroup analyses**

**S6A. Subgroup analysis (bipolar disorder) for efficacy as mean value for depressive symptoms at endpoint**

**
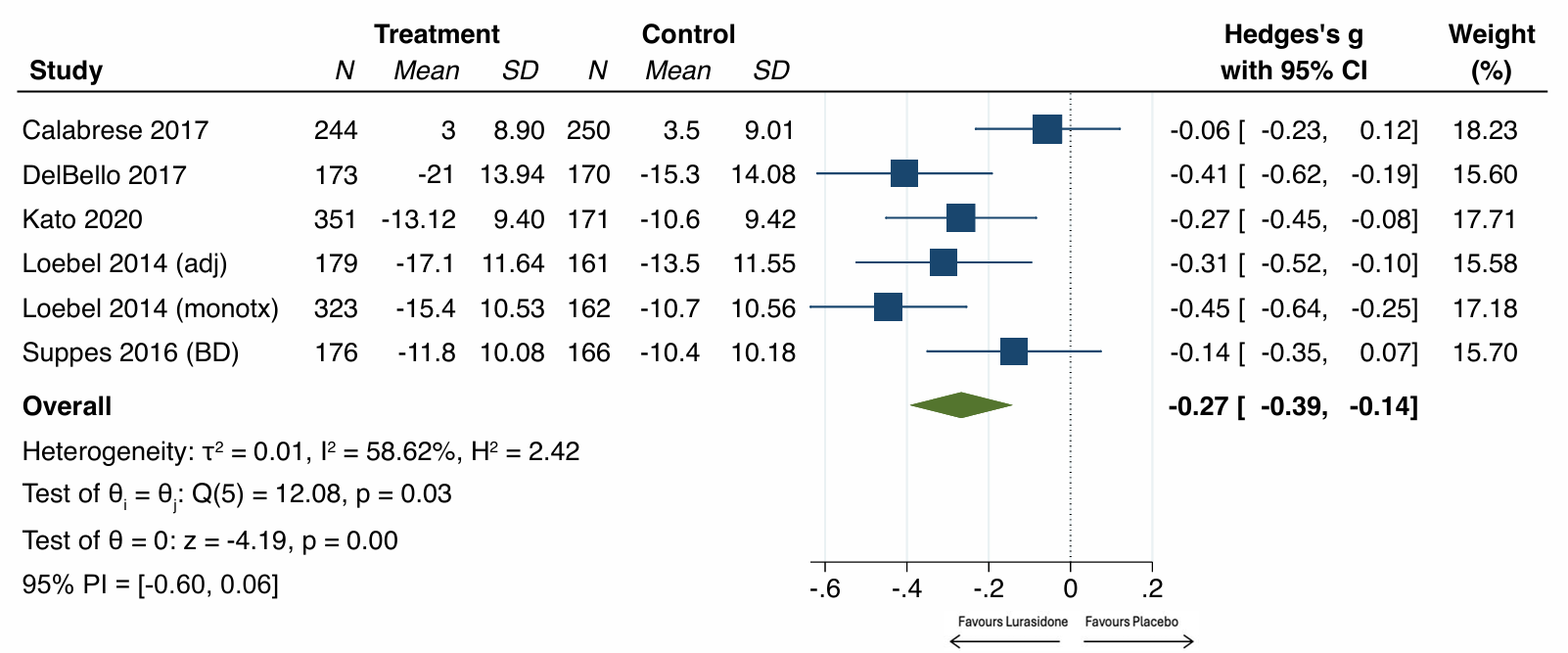
**

**S6B. Subgroup analysis (schizophrenia) for efficacy as mean value for depressive symptoms at endpoint**

**
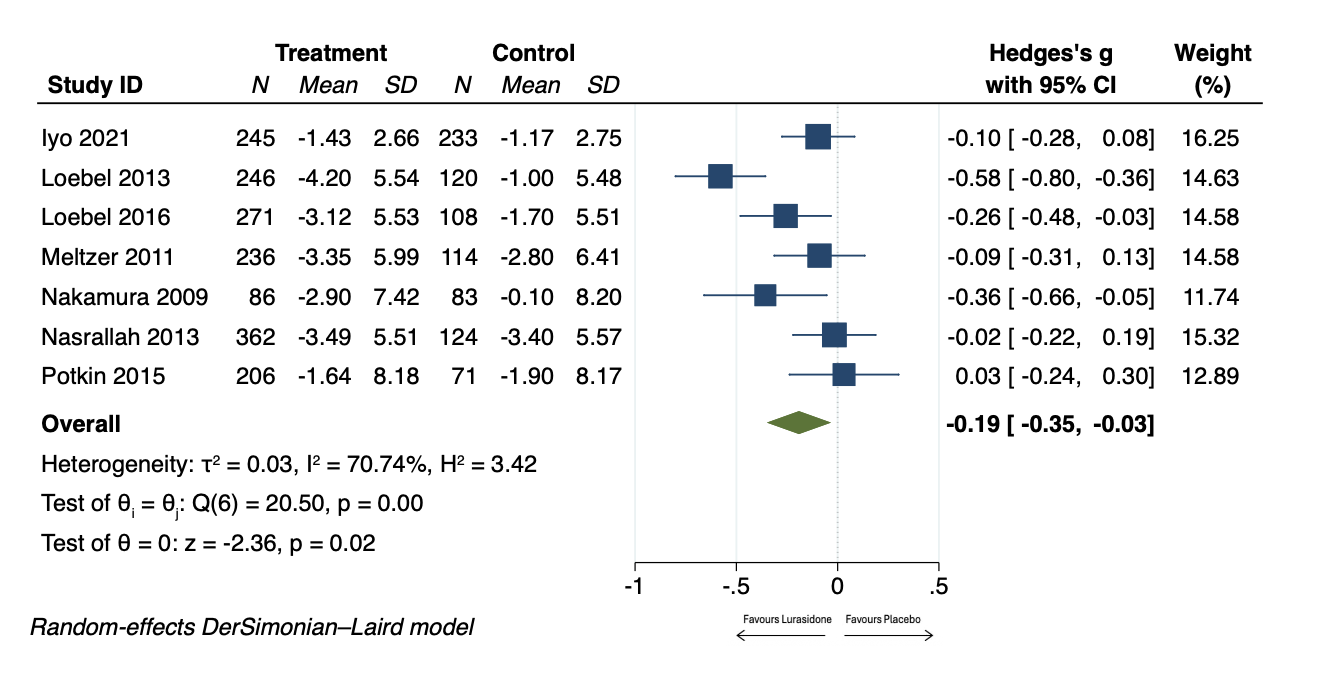
**

**Supplementary material S7. Safety analysis**

**
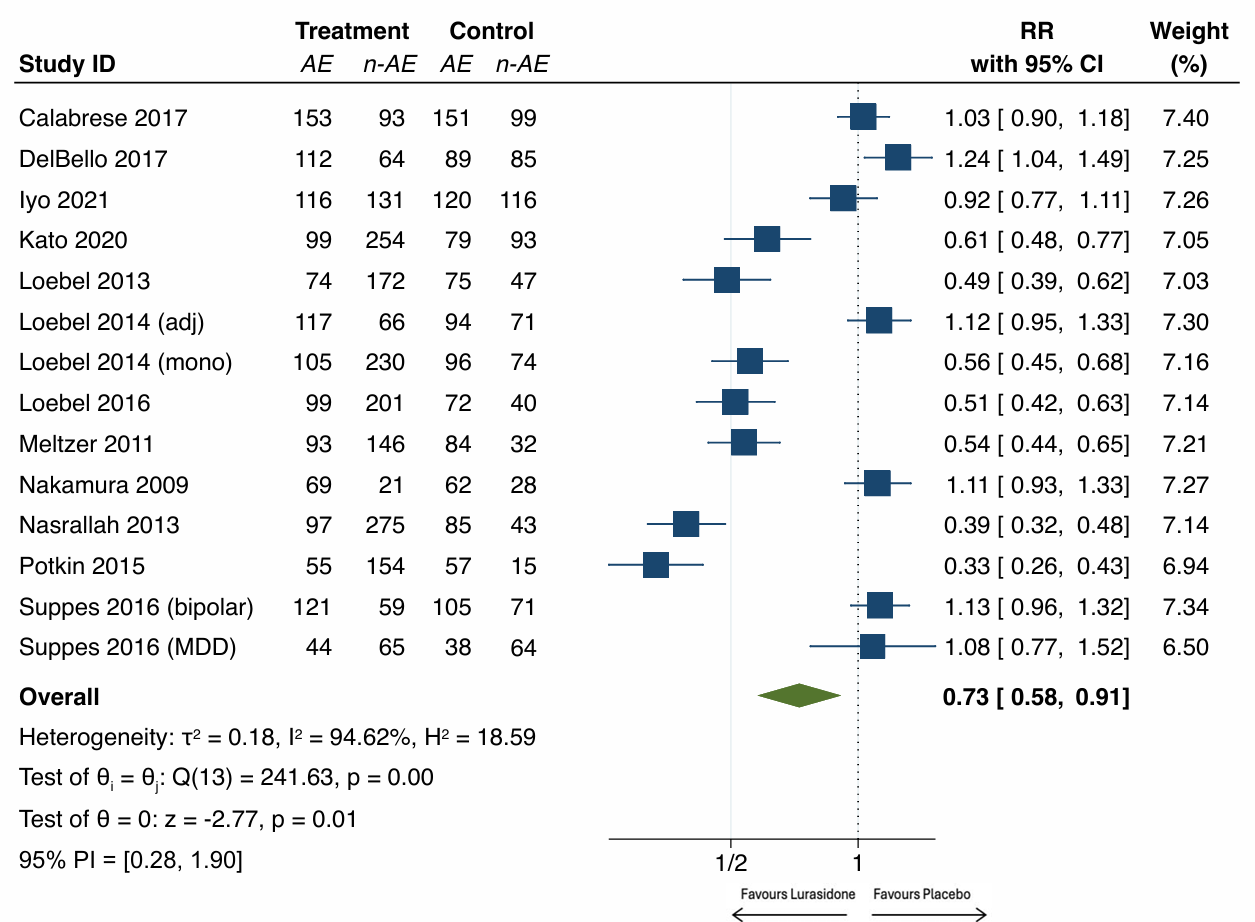
**

**Supplementary material S8. Assessment of publication bias.**

**S8A. Efficacy (depressive symptoms)**

*Regression-based Egger test for small study effects*

Random-effects model, DerSimonian–Laird

H0: beta1 = 0; no small-study effects

beta1 = -3.72

SE of beta1 = 3.118

z = -1.19

Prob > |z| = 0.2328

#### *Funnel plot*

**S8B. Acceptability (dropout due to any cause)**

#### *Regression-based Egger test for small study effects*

H0: beta1 = 0; no small-study effects

beta1 = -0.44

SE of beta1 = 1.973

z = -0.22

Prob > |z| = 0.8233

#### *Funnel plot*

**S8C. Tolerability (dropout due to adverse events)**

#### *Regression-based Egger test for small study effects*

H0: beta1 = 0; no small-study effects

beta1 = 1.21

SE of beta1 = 1.120

z = 1.08

Prob > |z| = 0.2782

#### *Funnel plot*

**S8D. Safety (number of participants with at least one adverse)**

#### *Regression-based Egger test for small study effects*

H0: beta1 = 0; no small-study effects

beta1 = -6.53

SE of beta1 = 4.169

z = -1.57

Prob > |z| = 0.1174

#### *Funnel plot*

**S9. RoB2 assessment of quality of trials**

**S9A. Risk of bias table, summary**


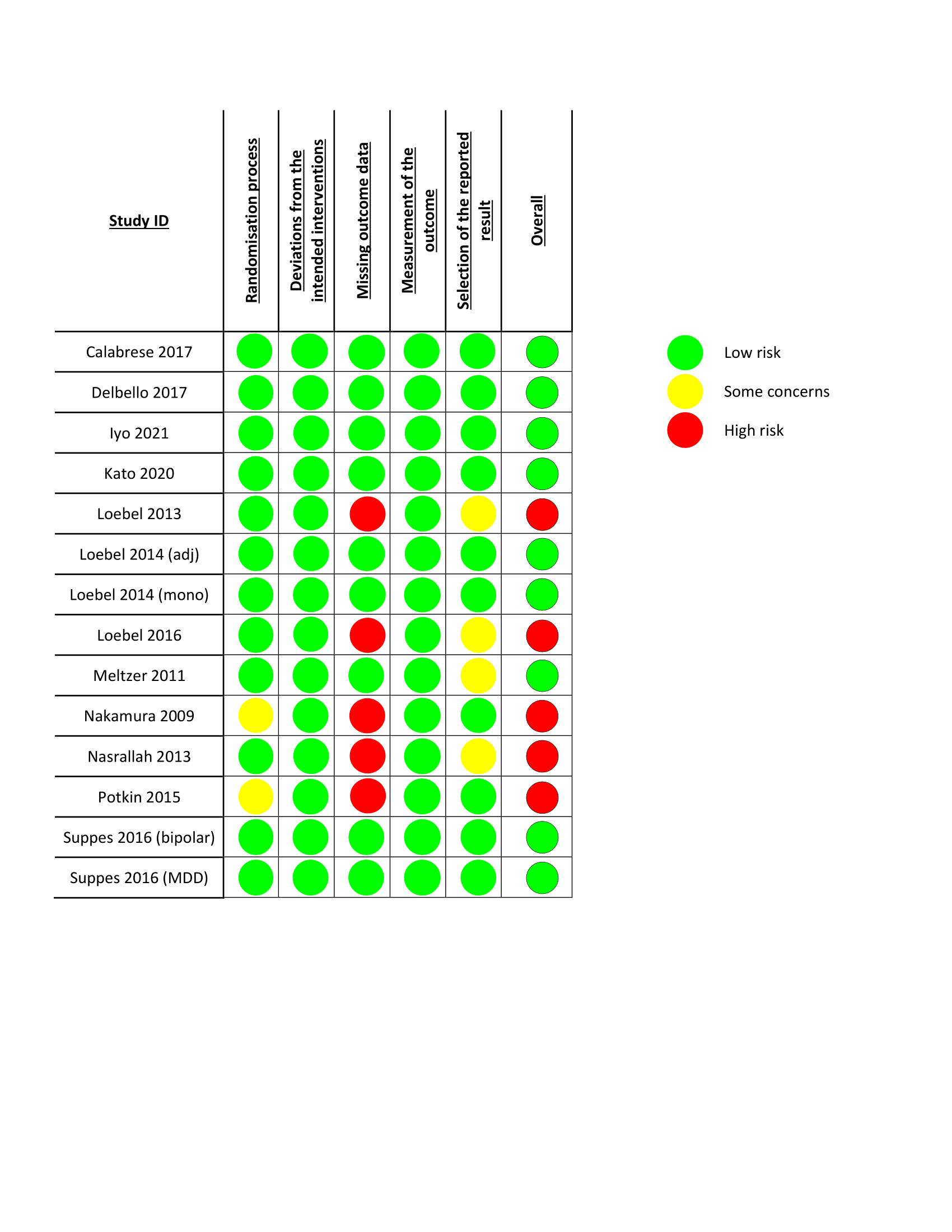


**S9B. Risk of bias table, noted
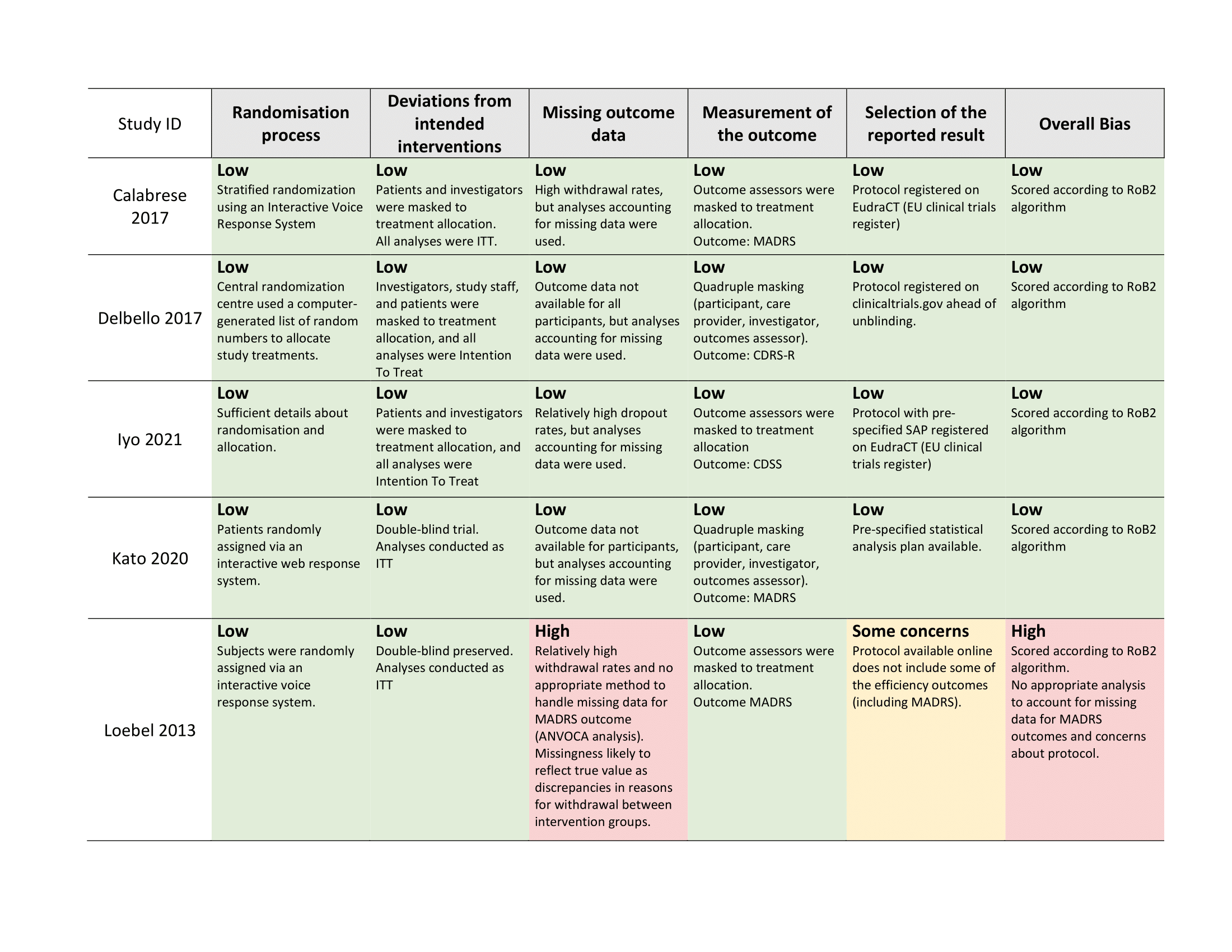
**


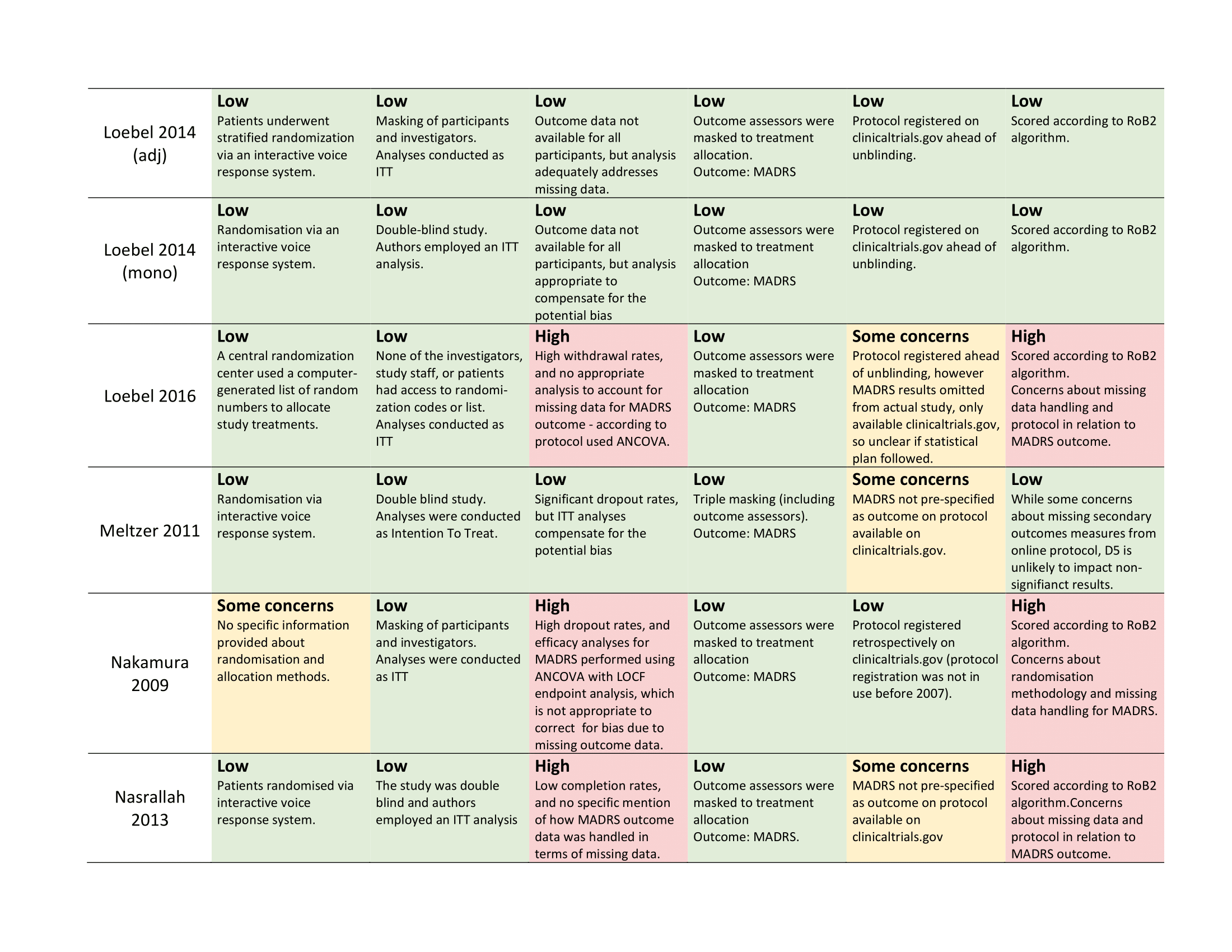

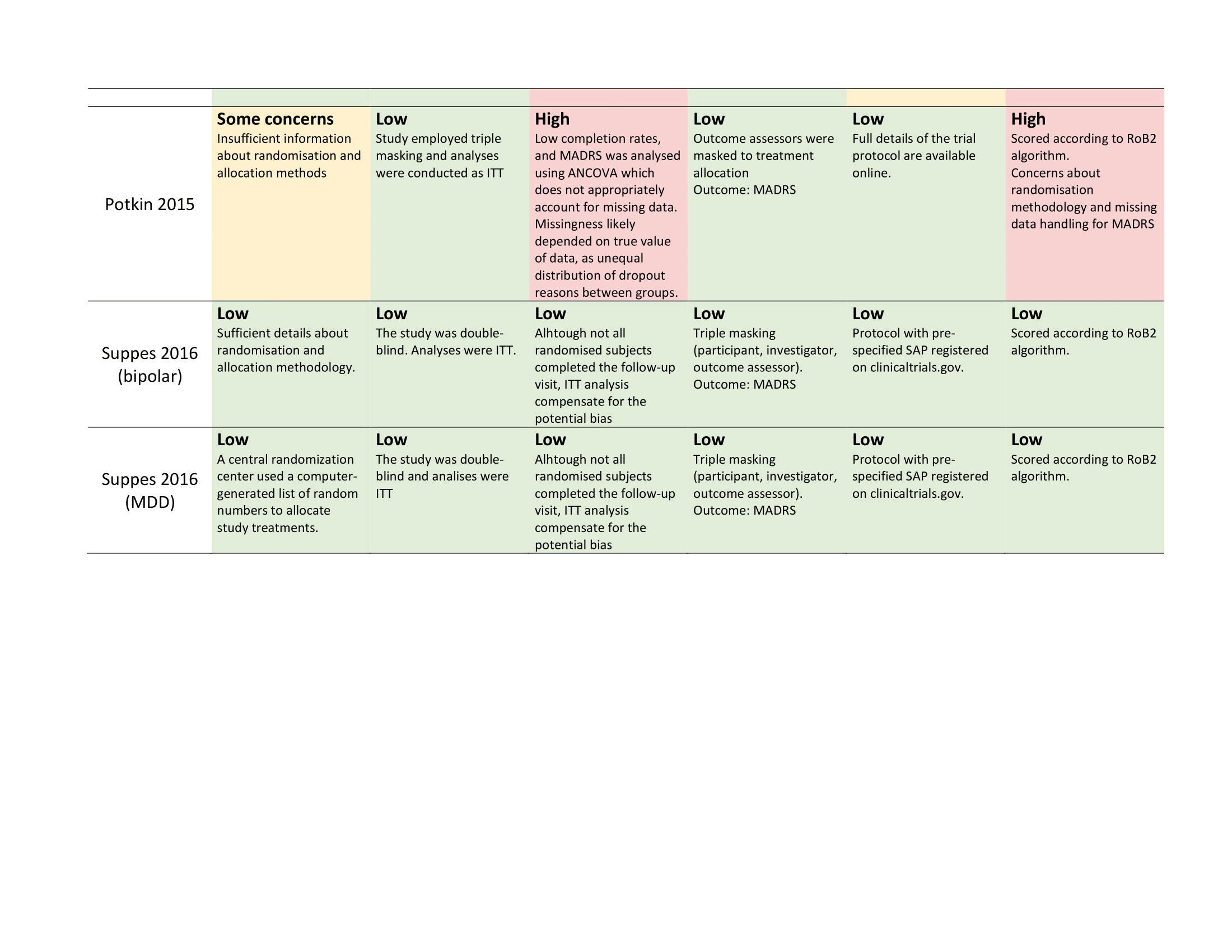


**S9C. Risk of bias table, across trials**

**S10. GRADE assessment of certainty of evidence**

| **Lurasidone compared to Control for Depressive symptoms** | | | | | | | | | | | |
| --- | --- | --- | --- | --- | --- | --- | --- | --- | --- | --- | --- |
| **Certainty assessment** | | | | | | | **Summary of findings** | | | | |
| **Participants (studies) Follow-up** | **Risk of bias** | **Inconsistency** | **Indirectness** | **Imprecision** | **Publication bias** | **Overall certainty of evidence** | **Study event rates (%)** | | **Relative effect (95% CI)** | **Anticipated absolute effects** | |
|  |  |  |  |  |  |  | **With Control** | **With Lurasidone** |  | **Risk with Control** | **Risk difference with Lurasidone** |
| **Efficacy (depressive symptoms) (follow-up: range 6 weeks to 28 weeks; assessed with: Any depression rating scale)** | | | | | | | | | | | |
| 5239 (14 RCTs) | serious^a^ | serious^b^ | not serious^c^ | not serious^d^ | none | ⨁⨁◯◯ Low | 2033 | 3206 | - | - | SMD **0.26 SD lower** (0.37 lower to 0.15 lower) |
| **Acceptability (dropout due to any cause) (follow-up: range 6 weeks to 28 weeks)** | | | | | | | | | | | |
| 5370 (14 RCTs) | not serious^e^ | very serious^f^ | not serious^c^ | not serious^g^ | none | ⨁⨁◯◯ Low | 603/2085 (28.9%) | 530/3285 (16.1%) | **RR 0.55** (0.43 to 0.71) | 603/2085 (28.9%) | **130 fewer per 1,000** (from 165 fewer to 84 fewer) |
| **Tolerability (dropout due to adverse events) (follow-up: range 6 weeks to 28 weeks)** | | | | | | | | | | | |
| 5370 (14 RCTs) | not serious^e^ | not serious^h^ | not serious^c^ | serious^i^ | none | ⨁⨁⨁◯ Moderate | 95/2085 (4.6%) | 111/3285 (3.4%) | **RR 0.74** (0.54 to 1.02) | 95/2085 (4.6%) | **12 fewer per 1,000** (from 21 fewer to 1 more) |
| **Safety (number of participants with at least one adverse event) (follow-up: range 6 weeks to 28 weeks)** | | | | | | | | | | | |
| 5370 (14 RCTs) | not serious^e^ | very serious^j^ | not serious^c^ | not serious^k^ | none | ⨁⨁◯◯ Low | 1207/2085 (57.9%) | 1354/3285 (41.2%) | **RR 0.73** (0.68 to 0.91) | 1207/2085 (57.9%) | **156 fewer per 1,000** (from 185 fewer to 52 fewer) |

**CI:** confidence interval; **RR:** risk ratio; **SMD:** standardised mean difference

#### Explanations

a. 1,677 participants (33.56% study weight) of the 5,239 total sample are from studies scored as high risk of bias due to missing outcome data

b. I2 = 72.07%

c. Transdiagnostic population (schizophrenia, bipolar disoder, major depressive disorder) as per study aim

d. OIS met (5,239 total sample), 95% CIs consistent with small to moderate beneficial effect size (SMD = -0.37 to -0.15)

e. No missing data for this outcome

f. I2 = 83.65%

g. OIS met (5,370 total sample), 95% CIs consistent with moderate beneficial effect size (RR = 0.43 to 0.71)

h. I2 = 22.25%

i. OIS met (5,370 total sample), 95% CIs crossing the line of null-effect and consistent with both a moderate beneficial effect size and a small harmful effect size (RR = 0.54 to 1.02)

j. I2 = 94.62%

k. OIS met (5,370 total sample), 95% CIs consistent with small to moderate beneficial effect size (RR = 0.68 to 0.91)

**Supplementary material S11. Summary of changes from protocol**

| **Element changed** | **Details of change** |
| --- | --- |
| **Title** | Changed to accurately reflect inclusion of meta-analysis |
| **Anticipated completion date** | Changed to accurately reflect completion timeline |
| **Planned subgroup analysis based on different endpoints** | We had planned to analyse data based on different endpoints: short term (<2 months), medium term (2-6 months), and long-term (>6 months). Since all but one trial ended at 6 weeks, we instead performed a sensitivity analysis excluding (*Calabrese 2017*) which had a duration of 28 weeks. |
| **Analysis of effects on negative symptoms in schizophrenia** | We had planned to analyse effects on negative symptoms in schizophrenia cohorts using the Positive and Negative Syndrome Scale (PANSS) negative subscale or any other standardised scale. This was not done as it was decided it would be beyond the scope of the paper, considering our search revealed many RCTs with PANSS as a primary outcome measure but no outcomes related to depression. |

**Supplementary material S12. Open-label extension studies of included trials**

| **Extension study ID** | **Original Included Study ID(s)** | **Efficacy population** | **Diagnosis** | **Intervention** | **Extension Length** | **Efficacy result** |
| --- | --- | --- | --- | --- | --- | --- |
| **Goldberg 2017** (1) | Suppes 2016b | 40 adults 18-75 years | MDD with mixed features | Lurasidone 20-60 mg/day | 3 months | Sustained recovery defined as both symptomatic remission (MADRS ≤ 12) and functional remission (all Sheehan Disability Scale [SDS] domain scores ≤3)  Sustained recovery rates for both months 1 and 3:  Lurasidone-lurasidone: 20.8%  Placebo-lurasidone: 12.5% |
| **Ketter 2016** (2) | Loebel 2014b | 301 adults 18-75 years | Bipolar I disorder, current major depressive episode | Lurasidone 20-120mg/day; concomitant mood stabilizer, benzodiazepine, or antidepressant use permitted. Additional antipsychotics prohibited. | 24 weeks | Change in MADRS scores, mean (SD):  Lurasidone-lurasidone: -20.1 (9.5) from double-blind baseline to month 6; -3.3 (9.7) from open-label baseline to month 6  Placebo-lurasidone: -20.1 (9.2) from double-blind to month 6; -7.6 (10.9) from open-label baseline to month 6 |
|  | Loebel 2014a  Suppes 2016a | 477 adults  18-75 years |  |  |  | Change in MADRS scores mean (SD):  Lurasidone-lurasidone: -18.7 (10.5) from double-blind baseline to month 6; -3.0 (10.7) from open-label baseline to month 6  Placebo-lurasidone: -18.5 (10.5) from double-blind to month 6; -5.5 (10.7) from open-label baseline to month 6 |
| **Pikalov 2017** (3) | Loebel 2014a  Loebel 2014b  Suppes 2016a | 122 18-month extension study | Bipolar I disorder, major depressive episode | Lurasidone 20-80mg; concomitant mood stabilizer or antidepressant use permitted | Additional 18-month extension of 6-month extension study (Ketter 2016) | Clinical response in global depression symptom severity defined as borderline-to-no symptoms (CGI-S ≤ 2); full symptom remission defined as (CGI-S = 1)  62.7% and 32.8% response and remission, respectively at open-label baseline  73.9% and 36.2% response and remission rates respectively at LOCF end point |
| **Ishigooka 2021** (4) | Kato 2020 | 413 adults 18-74 years | Bipolar I depression | Lurasidone 20-120mg/day; certain concomitant medication permitted, including lithium or valproate | 28 weeks | Change in MADRS score mean (SD) from open-label baseline to week 28:  Lurasidone-lurasidone: -5.1 (11.4)  Placebo-lurasidone: -7.6 (12.7) |
| **Higuchi 2021** (5) | Kato 2020 | 198 adults 18-74 years | Bipolar I disorder: either depressed episode (from Kato RCT) or newly recruited manic, hypomanic, or mixed episode | Lurasidone 20-120 mg/day; certain concomitant medication permitted, including lithium or valproate | 52 weeks | Change in MADRS score mean (SD) from open-label baseline to end-point (up to week 52):  Depressed group, lurasidone-lurasidone:  1.4 (12.9)  Depressed group, placebo-lurasidone: − 2.0 (14.7)  Non-depressed group: 2.5 (10.9) |
| **Delbello 2021** (6) | DelBello 2017 | 305 adolescents 10-17 years | Bipolar I disorder, major depressive episode | Lurasidone 20-80mg/day: certain concomitant medication permitted, not including mood stabilizer use | 2 years | CDRS-R change mean (SD) from baseline to week 104: -16.4 (13.24) |

**References**

1. Goldberg JF, Ng-Mak D, Siu C, Chuang CC, Rajagopalan K, Loebel A. Remission and recovery associated with lurasidone in the treatment of major depressive disorder with subthreshold hypomanic symptoms (mixed features): post-hoc analysis of a randomized, placebo-controlled study with longer-term extension. *CNS spectrums*. 2017;22(2): 220–227. https://doi.org/10.1017/S1092852917000025.

2. Ketter TA, Sarma K, Silva R, Kroger H, Cucchiaro J, Loebel A. LURASIDONE IN THE LONG-TERM TREATMENT OF PATIENTS WITH BIPOLAR DISORDER: A 24-WEEK OPEN-LABEL EXTENSION STUDY. *Depression and anxiety*. 2016;33(5): 424–434. https://doi.org/10.1002/DA.22479.

3. Pikalov A, Tsai J, Mao Y, Silva R, Cucchiaro J, Loebel A. Long-term use of lurasidone in patients with bipolar disorder: safety and effectiveness over 2 years of treatment. *International journal of bipolar disorders*. 2017;5(1). https://doi.org/10.1186/S40345-017-0075-7.

4. Ishigooka J, Kato T, Miyajima M, Watabe K, Masuda T, Hagi K, et al. Lurasidone in the Long-Term Treatment of Bipolar I Depression: A 28-week Open Label Extension Study. *Journal of affective disorders*. 2021;281: 160–167. https://doi.org/10.1016/J.JAD.2020.12.005.

5. Higuchi T, Kato T, Miyajima M, Watabe K, Masuda T, Hagi K, et al. Lurasidone in the long-term treatment of Japanese patients with bipolar I disorder: a 52 week open label study. *International journal of bipolar disorders*. 2021;9(1). https://doi.org/10.1186/S40345-021-00230-8.

6. Delbello MP, Tocco M, Pikalov A, Deng L, Goldman R. Tolerability, Safety, and Effectiveness of Two Years of Treatment with Lurasidone in Children and Adolescents with Bipolar Depression. *Journal of child and adolescent psychopharmacology*. 2021;31(7): 494–503. https://doi.org/10.1089/CAP.2021.0040.

**Supplementary material S13.**

**S13A. Efficacy Data Extraction Table**

| **Study ID** | **Time point** | **Scale** | **Analysis** | **Dose (lurasidone)** | **n (Lurasidone)** | | **Lurasidone baseline mean** | **Lurasidone baseline SD** | **Lurasidone mean change** | **Lurasidone SE** | **n (Placebo)** | | **Placebo baseline mean** | **Placebo baseline SD** | **Placebo mean change** | **Placebo SE** | **p-value (effect Size)** |
| --- | --- | --- | --- | --- | --- | --- | --- | --- | --- | --- | --- | --- | --- | --- | --- | --- | --- |
|  |  |  |  |  | **Randomised** | **Analysed** |  |  |  |  | **Randomised** | **Analysed** |  |  |  |  |  |
| **Calabrese 2017** | Survival-Week 28 | MADRS | Mean change | 20-80mg | 246 | 244 | 4 | 3.4 | 3 | 0.57 | 250 | 250 | 4.1 | 3.7 | 3.5 | 0.57 | ns |
| **DelBello 2017** | Week 6 | CDRS-R (total) | Mean change | 20-80mg | 176 | 173 | 59.2 | 8.24 | -21 | 1.06 | 174 | 170 | 58.6 | 8.26 | -15.3 | 1.08 | <0.0001 (0.45) |
| **Iyo 2021** | Week 6 | CDSS (total) | Mean change | 40mg | 247 | 245 | 4.2 | 3.8 | -1.43 | 0.17 | 236 | 233 | 4.2 | 3.8 | -1.17 | 0.18 | 0.256 (0.11) |
| **Kato 2020** | Week 6 | MADRS | Mean change | 20-60mg | 184 | 182 | 30.6 | 5.6 | -13.6 | 0.69 | 172 | 171 | 30.9 | 5.4 | -10.6 | 0.72 | 0.007 (0.33) |
|  |  |  |  | 80-120mg | 169 | 169 | 30.8 | 5.1 | -12.6 | 0.73 |  |  |  |  |  |  | 0.057 (0.22) |
| **Loebel 2013** | Week 6 | MADRS | Mean change | 80mg | 125 | 125 | 11.6 | 7.6 | -4 | 0.5 | 122 | 120 | 11.3 | 6.7 | -1 | 0.5 | <0.001 |
|  |  |  |  | 160mg | 121 | 121 | 11.2 | 7.8 | -4.4 | 0.5 |  |  |  |  |  |  | <0.001 |
| **Loebel 2014 (adj)** | Week 6 | MADRS | Mean change | 20-120mg | 183 | 179 | 30.6 | 5.3 | -17.1 | 0.87 | 165 | 161 | 30.8 | 4.8 | -13.5 | 0.91 | 0.005 (0.34) |
| **Loebel 2014 (mono)** | Week 6 | MADRS | Mean change | 20-60mg | 166 | 161 | 30.3 | 5 | -15.4 | 0.83 | 170 | 162 | 30.5 | 5 | -10.7 | 0.83 | <0.001 (51) |
|  |  |  |  | 80-120mg | 169 | 162 | 30.6 | 4.9 | -15.4 | 0.83 |  |  |  |  |  |  | <0.001 (51) |
| **Loebel 2016** | Week 6 | MADRS | Mean change | 20mg | 101 | 93 |  |  | -2 | 0.57 | 112 | 108 |  |  | -1.7 | 0.53 | 0.706 |
|  |  |  |  | 80-160mg | 199 | 178 |  |  | -3.7 | 0.41 |  |  |  |  |  |  | 0.003 |
| **Meltzer 2011** | Week 6 | MADRS | Mean change | 40mg | 120 | 118 | 10.8 | 7 | -3.5 | 0.5 | 116 | 114 | 10.6 | 6.1 | -2.8 | 0.6 | 0.324 |
|  |  |  |  | 120mg | 119 | 118 | 11.4 | 7.2 | -3.2 | 0.6 |  |  |  |  |  |  | 0.571 |
| **Nakamura 2009** | Week 6 | MADRS | Mean change | 80mg | 90 | 86 | 14.2 | 8 | -2.9 | 0.8 | 90 | 83 | 14.5 | 8.3 | -0.1 | 0.9 | 0.0187 (0.37) |
| **Nasrallah 2013** | Week 6 | MADRS | Mean change | 40mg | 125 | 121 | 11.2 | 6.4 | -3.3 | 0.5 | 128 | 124 | 11.9 | 6.8 | -3.4 | 0.5 | "NS" only |
|  |  |  |  | 80mg | 123 | 118 | 11.1 | 7.1 | -4.3 | 0.5 |  |  |  |  |  |  |  |
|  |  |  |  | 120mg | 124 | 123 | 11.3 | 7.3 | -2.9 | 0.5 |  |  |  |  |  |  |  |
| **Potkin 2015** | Week 6 | MADRS | Mean change | 20mg | 71 | 71 | 13.5 | 7.3 | -1.3 | 0.97 | 72 | 71 | 14.7 | 8.6 | -1.9 | 0.97 | 0.62 |
|  |  |  |  | 40mg | 67 | 65 | 13.1 | 7.5 | -1.1 | 1.02 |  |  |  |  |  |  | 0.565 |
|  |  |  |  | 80mg | 71 | 70 | 13.6 | 8 | -2.5 | 0.98 |  |  |  |  |  |  | 0.668 |
| **Suppes 2016 (bipolar)** | Week 6 | MADRS | Mean change | 20-120mg | 180 | 176 | 29.1 | 4.9 | -11.8 | 0.76 | 176 | 166 | 29.1 | 4.7 | -10.4 | 0.79 | 0.176 (0.16) |
| **Suppes 2016 (MDD)** | Week 6 | MADRS | Mean change | 20-60mg | 109 | 108 | 33.2 | 4.3 | -20.5 | 1 | 102 | 100 | 33.3 | 4 | -13 | 1 | <0.001 (-0.8) |

**S13B. Safety Data Extraction Table**

| **Study ID** | **Dose (Lurasidone)** | **n (Lurasidone)** | **Lurasidone - any adverse events N (%)** | **Lurasidone - resulting in withdrawal** | **Lurasidone - withdrawal for any reason** | **n (Placebo)** | **Placebo - any adverse events N (%)** | **Placebo - resulting in withdrawal** | **Placebo - withdrawal for any reason** | **Common adverse events** |
| --- | --- | --- | --- | --- | --- | --- | --- | --- | --- | --- |
| **Calabrese 2017** | 20-80mg | 246 | 153 (62.2%) | 8 (3.3%) | 80 (32.5%) | 250 | 151 (60.4%) | 5 (2%) | 100 (40%) | Weight increase, headache, parkinsonism, insomnia |
| **DelBello 2017** | 20-80mg | 175 | 112 (64%) | 3 (1.7%) | 14 (8%) | 172 | 89 (51.7%) | 3 (1.7%) | 18 (10.3%) | Nausea, somnolence, weight increase, vomiting, dizziness, insomnia, decreased appetite |
| **Iyo 2021** | 40mg | 247 | 116 (47%) | 14 (5.66%) | 48 (19.43%) | 235 | 120 (51.1%) | 15 (6.38%) | 60 (25.53%) | Akathisia, dizziness, somnolence, abdominal discomfort, asthenia |
| **Kato 2020** | 20-60mg | 184 | 98 (53.3%) | 6 (3.26%) | 27 (14.67%) | 172 | 79 (45.9%) | 7 (4.07%) | 33 (19.18%) | Akathisia, nausea, somnolence, parkinsonism, nasopharyngitis |
|  | 80-120mg | 169 | 100 (59.2%) | 16 (9.46%) | 32 (18.93%) |  |  |  |  |  |
| **Loebel 2013** | 80mg | 125 | 72 (57.6%) | 5 (4%) | 26 (28.8%) | 121 | 75 (62%) | 5 (4.1%) | 48 (39.3%) | Headache, insomnia, akathisia, nausea, vomiting, anxiety |
|  | 160mg | 121 | 76 (62.8%) | 4 (3.3%) | 28 (23.1%) |  |  |  |  |  |
| **Loebel 2014 (adjunctive)** | 20-120mg | 183 | 117 (63.9%) | 11 (6.01%) | 40 (21.85%) | 163 | 94 (57.7%) | 13 (7.97%) | 29 (17.79%) | Nausea, somnolence, tremor, akathisia, insomnia |
| **Loebel 2014 (monotherapy)** | 20-60mg | 164 | 101 (61.6%) | 11 (6.62%) | 43 (25.9%) | 168 | 96 (57.1%) | 11 (6.47%) | 43 (25.29%) | Nausea, headache, akathisia, somnolence, sedation, dry mouth, vomiting |
|  | 80-120mg | 167 | 108 (64.7%) | 10 (5.91%) | 43 (25.29%) |  |  |  |  |  |
| **Loebel 2016** | 20mg | 101 | 55 (54.5%) | 2 (1.98%) | 27 (26.7%) | 112 | 72 (64.3%) | 8 (7.14%) | 42 (37.5%) | Insomnia, headache, anxiety, agitation, akathisia, nausea, somnolence, sedation |
|  | 80-160mg | 198 | 98 (49.5%) | 8 (4.04%) | 53 (26.8%) |  |  |  |  |  |
| **Meltzer 2011** | 40mg | 119 | 90 (75.6%) | 8 (6.66%) | 43 (35.83%) | 116 | 84 (72.4%) | 10 (8.62%) | 45 (38.79%) | Akathisia, somnolence, sedation, parkinsonism |
|  | 120mg | 118 | 97 (82.2%) | 14 (11.76%) | 53 (44.53%) |  |  |  |  |  |
| **Nakamura 2009** | 80mg | 90 | 69 (76.7%) | 6 (6.7%) | 38 (42.2%) | 90 | 62 (68.9%) | 1 (1.1%) | 43 (47.8%) | Gastrointestinal (nausea, constipation, vomiting, dyspepsia) |
| **Nasrallah 2013** | 40mg | 124 | 96 (77.4%) | 5 (4%) | 41 (32.8%) | 127 | 85 (66.9%) | 3 (2.3%) | 55 (43%) | Akathisia, headache, somnolence, nausea, sedation |
|  | 80mg | 121 | 90 (74.4%) | 8 (6.5%) | 37 (30.1%) |  |  |  |  |  |
|  | 120mg | 124 | 106 (85.5%) | 7 (5.6%) | 39 (31.5%) |  |  |  |  |  |
| **Potkin 2015** | 20mg | 71 | 53 (74.6%) | 1 (1%) | 44 (62%) | 72 | 57 (79.2%) | 4 (6%) | 36 (50%) | Sedation, dyspepsia, nausea, akathisia |
|  | 40mg | 67 | 57 (85.1%) | 8 (12%) | 39 (58%) |  |  |  |  |  |
|  | 80mg | 71 | 56 (78.9%) | 7 (10%) | 40 (56%) |  |  |  |  |  |
| **Suppes 2016 (bipolar)** | 20-120mg | 177 | 121 (68.4%) | 11 (6.1%) | 32 (17.8%) | 171 | 105 (61.4%) | 5 (2.8%) | 36 (20.5%) | Akathisia, somnolence, extrapyramidal symptoms, nausea, diarrhoea |
| **Suppes 2016 (MDD)** | 20-60mg | 109 | 44 (40.4%) | 3 (2.8%) | 7 (6.4%) | 100 | 38 (38%) | 5 (4.9%) | 15 (14.7%) | Nausea, somnolence, dizziness, akathisia, abdominal discomfort, dry mouth, parkinsonism |
